# Shedding of cancer susceptibility candidate 4 by the convertases PC7/furin unravels a novel secretory protein implicated in cancer progression

**DOI:** 10.1038/s41419-020-02893-0

**Published:** 2020-08-20

**Authors:** Stéphanie Duval, Afnan Abu-Thuraia, Islam E. Elkholi, Rui Chen, Deeptee Seebun, Janice Mayne, Jean-François Côté, Daniel Figeys, Nabil G. Seidah

**Affiliations:** 1 Laboratory of Biochemical Neuroendocrinology, Montreal Clinical Research Institute (IRCM; Affiliated to the University of Montreal), 110 Pine Ave West, Montreal, QC H2W1R7 Canada; 2 Laboratory of Cytoskeletal Organization and Cell Migration, Montreal Clinical Research Institute (IRCM; affiliated to the University of Montreal), 110 Pine Ave West, Montreal, QC H2W1R7 Canada; 3 Ottawa Institute of Systems Biology and Department of Biochemistry, Microbiology and Immunology, Faculty of Medicine, University of Ottawa, Ottawa, ON K1H 8M5 Canada

**Keywords:** Biochemistry, Proteases

## Abstract

The proprotein convertases (PCs) are responsible for the maturation of precursor proteins, and are involved in multiple and critical biological processes. Over the past 30 years, the PCs have had great translational applications, but the physiological roles of PC7, the seventh member of the family, are still obscure. Searching for new substrates of PC7, a quantitative proteomics screen for selective enrichment of *N*-glycosylated polypeptides secreted from hepatic HuH7 cells identified two human type-II transmembrane proteins of unknown function(s): Cancer Susceptibility Candidate 4 (CASC4) and Golgi Phosphoprotein of 130 kDa (GPP130/GOLIM4). Concentrating on CASC4, its mutagenesis characterized the PC7/Furin-shedding site to occur at KR_66_↓NS, in HEK293 cells. We defined PC7 and Furin trafficking and activity, and demonstrated that CASC4 shedding occurs in acidic endosomes and/or in the trans-Golgi Network. Our data unraveled a cancer-protective role for CASC4, because siRNA silencing of endogenous CASC4 expression in the invasive triple-negative breast cancer human cell line MDA-MB-231 resulted in a significantly increased cellular migration and invasion. Conversely, MDA-MB-231 cells stably expressing CASC4 exhibited reduced migration and invasion, which can be explained by an increased number of paxillin-positive focal adhesions. This phenotypic cancer-protective role of CASC4 is reversed in cells overexpressing an optimally PC7/Furin-cleaved CASC4 mutant, or upon overexpression of the N-terminally convertase-generated membrane-bound segment. This phenotype was associated with increased formation of podosome-like structures, especially evident in cells overexpressing the N-terminal fragment. In accord, breast cancer patients’ data sets show that high *CASC4* and *PCSK7* expression levels predict a significantly worse prognosis compared to high *CASC4* but low *PCSK7* levels. In conclusion, CASC4 shedding not only disrupts its anti-migratory/invasive role, but also generates a membrane-bound fragment that drastically modifies the actin cytoskeleton, resulting in an enhanced cellular migration and invasion. This phenotype might be clinically relevant in the prognosis of breast cancer patients.

## Introduction

The proprotein convertases (PCs) constitute a family of nine serine secretory proteases that regulate diverse biological processes in both health and disease states^1^. By irreversible proteolysis, PCs are responsible for the activation or inactivation of a variety of precursor proteins, such as growth factors, hormones, receptors and adhesion molecules^1^. Such cleavage or shedding events may also result in the generation of cleaved entities with distinct novel functions. The first seven PCs cleave (↓) precursor proteins at specific single or paired basic amino acid (aa) within the motif (R/K)-(2X_*n*_)-(R/K)↓, where X_*n*_ = 0–3 spacer aa^2^. Because of their roles in the processing of many critical secretory substrates, e.g., activation of TGF-β^3^ and matrix metalloproteases^4^, PCs, such as Furin, PC5, PACE4, and PC7 were implicated in cancer/metastasis^5–7^.

The seventh member of the family (PC7; gene *PCSK7*) is a ubiquitously expressed protease that often shares substrates with other PCs, especially Furin^1,8^. Recent work by our group identified the type-II transmembrane human transferrin receptor 1 (TfR1) as the first PC7-specific substrate^9^. Even though both Furin and PC7 are enriched in the TGN, Furin cleaves its substrates mostly in the TGN, cell surface or internalized endosomes^10,11^. In contrast, PC7 cleaves its only known specific substrate hTfR1 following its internalization from the cell surface into early endosomes^9,12^. To better understand the PC7 biology and pathophysiology, we undertook an unbiased quantitative proteomics screen of *N*-glycosylated secreted products from hepatic HuH7 cells overexpressing PC7. This screen led us to identify two shed type-II transmembrane proteins of unknown biological functions: Cancer Susceptibility Candidate 4 (CASC4)^13^, and Golgi Phosphoprotein of 130 kDa (GPP130/GOLIM4)^14^. *CASC4* was originally identified in a breast cancer screen in the context of HER2^**+**^ overexpression^13^. More recently, *CASC4* was also shown to be aberrantly spliced in breast cancer^15^ and glioblastoma^16^, however, the functional consequences of the spliced isoforms were not defined. In addition, a significant increase in secreted (shed) sCASC4 was found upon analysis of the *N*-glycosylated secretome from highly metastatic breast cancer cell lines^17^.

In this study, we demonstrated that PC7 and Furin specifically shed CASC4 in post-ER acidic compartments, generating an N-terminal membrane-bound domain (NTD) and a secreted C-terminal fragment. Using triple-negative breast cancer MDA-MB-231 cells stably expressing CASC4 or its selected mutants, we demonstrated that wild type (WT) CASC4 enhances cell adhesion by increasing the number of focal adhesions (FA) and actin stress fibers, and that its shedding by PC7/Furin abrogates this phenotype. We then showed that the PC7/Furin-generated NTD induced the formation of podosome-like structures, implicated in invasion^18,19^. These results provide a novel unique mechanistic rationale for the functions of CASC4 and its shedding by PCs, which impact cellular migration and invasion.

## Materials and methods

### Glyco-proteomic analysis of secretome from HEK293 and HuH7 cells overexpressing PC7

#### Transient transfection

HEK293 cells (obtained from ATCC) and HuH7 (obtained from the JCRB) were maintained in 5% CO_2_ at 37 °C were seeded at 6 × 10^5^ cells in 6-well plates and grown to 80% confluency in Dulbecco’s modified Eagle medium (DMEM; Invitrogen) supplemented with 10% (v/v) fetal bovine serum (FBS; Invitrogen), 1 mm sodium pyruvate (Life Technologies) and 28 μg/ml gentamycin (Millipore-Sigma). Cells were transiently transfected with plasmid pIRES2 containing cDNA encoding human PC7 at 2 μg and as control pIRES2 empty vector. Transfections were carried out following Lipofectamine 3000 (Invitrogen) recommended protocols with a 1 μg:1 μl ratio of DNA to Lipofectamine reagent in OPTI-MEM media (Invitrogen) for HEK293 cells and 1:4 for HuH7 cells. Six hours post transfection, media was replaced by 2 ml of fresh OPTI-MEM media. At 24 h post media change, spent media was collected, centrifuged at 16,000 × *g* for 2 min to remove cellular debris and supernatants stored at −80 °C.

#### Enrichment of secreted glycoproteome

Spent media from transient transfections of PC7 and empty vector were concentrated and equilibrated in 8 M urea by ultracentrifugation using Amicon Ultra-15 centrifugal filter units (3 kDa cut-off, Millipore-Sigma). A total of 500 ug of proteins were used for glycoprotein enrichment. Proteins were digested with trypsin as described in ref. ^20^. Briefly, proteins were reduced with 10 mM dithiothreitol (DTT) at 56 °C for 45 min, alkylated with 20 mM iodoacetamide (IAA) at room temperature for 1 h and digested with trypsin at a 1:50 ratio at 37 °C overnight. Glycopeptides were enriched by hydrophilic interaction chromatography solid phase extraction (HILIC-SPE) as described in ref. ^20^. Following capture, and washes to remove non-glycosylated peptides, the enriched glycopeptides were eluted from column and dried by vacuum centrifugation. The enriched fraction was deglycosylated using 50 units of PNGaseF (New England Biolabs) in 50 μl 100 mM ammonium bicarbonate at 37 °C overnight.

#### LC–MS/MS analysis and database search

Deglycosylated peptides were analyzed with an HPLC-MS/MS as per ref. ^20^, using Q Exactive mass spectrometer (ThermoFisher Scientific Inc.) (ThermoFisher). The instrument method consisted of one full MS scan from 300 to 1800 *m*/*z* followed by data-dependent MS/MS scan of the 12 most intense ions, a dynamic exclusion repeat count of 2, and repeat exclusion duration of 30. Data files were processed with MaxQuant (1.2.2.5). The resulting precursor masses were matched to the IPI human database (version 3.68, 87,061 entries), and included the standard MaxQuant contaminant database. Mass tolerances were 6 ppm and 0.05 Da for the precursor and fragment, respectively. Enzyme specificity was set as KR/P, and a maximum of two missed cleavages was allowed. Cysteine residue was set as a static modification of 57.0215 Da, and the methionine oxidation and asparagine deamination were set as a variable modification of 15.9949 and 0.9840 Da, respectively. The false discovery rate cut-offs for both peptides and proteins were set at 1%. The protein group file was imported into Perseus (version 1.2.0.17) where identifications from contaminants and reversed databases were removed. Label free quantification was carried out and significant changes in proteins were determined by two-sided *t*-tests.

### Plasmids

Human GPP130 WT (ThermoScientific, Open Bioscience), CASC4-WT (ThermoScientific, Open Bioscience), and its mutants (R60A, R62A, K65A, R66A, AA_65/66_, NTD, SP-ΔTM-CASC4, and 5REL) were subcloned, with a V5-tag at the C-terminus into pIRES2-EGFP vector (Clontech). All constructions (human transferrin receptor 1), human Furin, mouse PC5A, mouse PC5B, human PACE4, full-length human PC7, soluble rat PC7, soluble human PC7, and Sar1P-(H79G) were cloned in pIRES2-EGFP vector (Clontech).

### Cell culture, transfections, and cell treatments

HEK293 cells were grown in Dulbecco’s modified Eagle’s medium (DMEM, Invitrogen) with 10% fetal bovine serum (FBS, Invitrogen), CHO-ldlD and CHO-K1 cells were grown in DMEM/F12 medium with 10% FBS, MDA-MB-231 cells were grown in Dulbecco’s modified Eagle’s medium (DMEM, Invitrogen) with 10% fetal bovine serum (FBS, Invitrogen), MCF10a cells were grown in MEGM Mammary Epithelial Cell Growth Medium BulletKit from Lonza (Catalog #: CC-3150) + 5% horse serum. All cells were maintained at 37 °C under 5% CO. HEK293 cells were co-transfected with equimolar quantities (0.5 μg) of each plasmid using Jetprime Polyplus, CHO-ldlD cells were transfected with equimolar quantities (1.0 μg) of each plasmid using FuGene HD, using manufacturer’s instructions. MDA-MB-231 cells were transfected using GenJet^TM^ In Vitro DNA Transfection Reagent for MDA-MB231 Cells (SignaGen Laboratories) with equimolar quantities of plasmids (1.5–2 μg) using manufacturer’s instructions. At 24 h post transfection, cells were washed in serum-free medium followed by an additional 20 h alone or in incubation with 2.5 μg/ml brefeldin A (BFA; Calbiochem), Dynasore (80 µM; Sigma), decanoyl-RVKR-CMK (75 µM; Bachem), hexapeptide (D-Arg)_6_ (10 µM; EMD Chemicals), or 20 mM ammonium chloride (NH_4_Cl; Sigma). For Endo H and PGNase F treatments, cell lysates were incubated with endoglycosidase H (endo H) or Peptide-*N*-Glycosidase F (PGNase F) for 1 h at 37 °C (New England Biolabs), cells and media were collected for western blot analysis. All experiments were repeated at last three times and center values represent the mean ± standard error from the mean.

### siRNAs and quantitative RT-qPCR

A pool of four siRNAs against human CASC4 and a scrambled siRNA (Dharmacon; siGENOME SMARTpool) were transfected with a final 100 nM concentration, using DhamaFECT4 transfection reagent (Dharmacon), using the manufacturer’s protocol. Total RNA extraction was performed using 1 ml Trizol reagent (Invitrogen) according to the manufacturer’s instructions. Real-time PCR was carried out using Viia7 System (Applied Biosystems). Reactions were run in duplicate for each independent experiment. Human TATA-box binding protein (hTBP) gene was used as an internal control to normalize the variability in expression levels. Supplementary Table S1 summaries oligonucleotide sequences used for human *CASC4*, human *PCSK7* and human *Furin*. All experiments were repeated at last three times and center values represent the mean ± standard error from the mean.

### Affinity-precipitation of GTP-Rho/Cdc42

MDA-MB-231 cells were washed with ice-cold Phosphate-buffered saline and lysed in Cytoskeleton Lysis Buffer (50 mM Tris pH 7.5, 10 mM MgCl_2_, 0.5 M NaCl, and 2% Igepal). Cell lysates were clarified by centrifugation at 10,000 × *g* at 4 °C for 1 min, and equal protein concentrations from the different cell lysates were incubated with GST–RBD (25 µg) or GST-PAK (10 µg) beads at 4 °C for 60 min. The beads were washed two times with washing buffer (25 mM Tris pH 7.5, 30 mM MgCl2, 40 mM NaCl). Bound Rho/Cdc42 proteins were detected by western blotting using a monoclonal antibody against RhoA/Cdc42 (Cytoskeleton). Densitometry analysis was performed using Image J software (National Institutes of Health). The amount of RBD-bound Rho was normalized to the total amount of Rho/Cdc42 in cell lysates for the comparison of Rho activity (level of GTPbound Rho/Cdc42) in the different samples. All experiments were repeated at last three times and center values represent the mean ± standard error from the mean.

### Western blot analysis and antibodies

Cells were lysed in cold Radio-Immunoprecipitation Assay (RIPA) buffer (100 mM Tris-HCl pH 8, 300 mM NaCl, 0.2% SDS, 2% NP-40, 1% Na deoxycholate) containing protease inhibitors (Roche Applied Bioscience). Proteins were analyzed by SDS-PAGE on 8–12% Tris-Glycine and transferred on a nitrocellulose membrane (GE Healthcare Life Science, No. 10600003) followed by 1 h blocking in Li-Cor blocking buffer (Li-Cor) or in 5% milk in TBST-T. Membranes were then incubated with primary antibody overnight. Proteins were visualized using mouse anti-V5 (1/2000, Invitrogen), rabbit anti-PC7 (1:10,000, homemade or 1:5000 Cell Signaling Technologies), Furin (1:5000, Invitrogen), rabbit anti β-actin (1:5000, Sigma-Aldrich), anti-Tubulin (1:5000, Sigma-Aldrich), p-paxillin(Y118) (ThermoFisher), paxillin (Transduction Laboratories), CASC4 (1:500, Abcam), Cdc42 (1:250, Cytoskeleton), RhoA (1:500; Cytoskeleton), or a horseradish peroxidase (HRP)-conjugated mAb V5 (1:10,000; Sigma-Aldrich), or anti-Flag M2 HRP (1:3000; Sigma-Aldrich). Bound primary antibodies were detected with corresponding species-specific fluorescent anti-mouse antibody 680 (Mandel) (1:10,000) or anti-rabbit Ab 800 (Mandel), and revealed using LiCor Bioscience, or with corresponding species-specific HRP-labeled secondary antibodies (1:10,000; Invitrogen) and revealed by enhanced chemiluminescence (ECL; Amersham). Quantifications were done using Image Studio Lite v.4.0 and Image J software (National Institutes of Health). All experiments were repeated at last three times and center values represent the mean ± standard error from the mean.

### Boyden migration and invasion assays

For migration assays, 1 × 10^5^ cells were seeded into a transwell (6.5 mm, Polycarbonate membrane 0.8 µM, VWR) and allowed to migrate towards 10% FBS DMEM medium into the lower chamber as a chemoattractant during 6 h. For invasion, 5 × 10^4^ cells were seeded into a Matrigel matrix (Corning) and allowed to invade during 16 h towards 10% FBS DMEM medium into the lower chamber. After migration/invasion, cells were aspirated from the transwell and the membranes were washed before fixation by 4% paraformaldehyde (ThermoScientific) for 10 min. Following fixation, membranes were washed three times and mounted onto a microscope slide (Fisher Scientific) with ProLong Gold antifade with DAPI (Invitrogen) to stain for nucleus. The fixed membranes were analyzed on a DMRB microscope at ×20 amplification, ten pictures were taken per condition and nuclei counted. All experiments were repeated at last three times and center values represent the mean ± standard error from the mean.

### Wound healing assay

Confluent monolayer of cells was scratched with a 200 µl-pipette tip to generate a scratch wound. To evaluate the distance traveled by the cells, pictures were taken with a Leica microscope at 6 and 12 h post-scratch. Images were analyzed using Image J software (National Institutes of Health). All experiments were repeated at last three times and center values represent the mean ± standard error from the mean.

### Microscopy analysis and antibodies

For F-actin staining, cells were seeded on Fibronectin (Sigma) coated (20 µg/ml) glass coverslip into 24 well plates and grown for 48 h. Cells were then fixed with warm paraformaldehyde (4%) for 20 min and permeabilized with PBS 1× + Triton 0.1%. Followed permeabilization, cells were stained for primary antibodies: paxillin (Transduction laboratories), anti-LDLR (R&D systems), anti-Golgin-97 (Santa Cruz Biotechnology), V5 (Invitrogen), and TKS5 (Millipore-Sigma) for 1 h followed by fluorescent corresponding secondary antibodies or fluorescent coupled 555-Phalloidin to stain for F-actin for 30 min. Coverslips were then mounted on microscope slide (Fisher Scientific) with ProLong Gold antifade with DAPI (Invitrogen) to stain for nucleus. Colocalization of fluorescently labeled protein was quantified with IMARIS analysis software (8.2.1) along with aXTension script named Colocalize Spots. We used the same approach as mentioned in Rajan et al.^21^. Positive signals were found using the Imaris function spots from each fluorescent marker images. The spot diameter used was 1.2 μm with the same quality factor for each image. The Colocalize Spots script considers colocalization between two spots when their center to center distance is equal of inferior to 0.8 μm. FA quantifications were done using Image J software (National Institutes of Health) with a program previously described^22^. All experiments were repeated at last three times and center values represent the mean ± standard error from the mean.

### Clinical data analysis

METABRIC^23,24^ and TCGA^25^ data sets were accessed through the cBioPortal online platform^26,27^. To investigate the correlation between the CASC4 and PCSK7 mRNA levels, the plot tool was used. To correlate the association of CASC4 and PCSK7 mRNA expression levels with the survival rate in the METABRIC data set, the expression level values of both genes (in *z*-scores) were downloaded for the whole data set. Threshold of *z*-score = /higher than 1 is used to identify patients with high expression of PCSK7 or CASC4 and threshold of *z*-score = /lower than −1 is used to identify patients with low expression of PCSK7.

## Results

### Mass spectrometry identifies two novel type-II transmembrane proteins cleaved by PC7

**Fig. 1:**
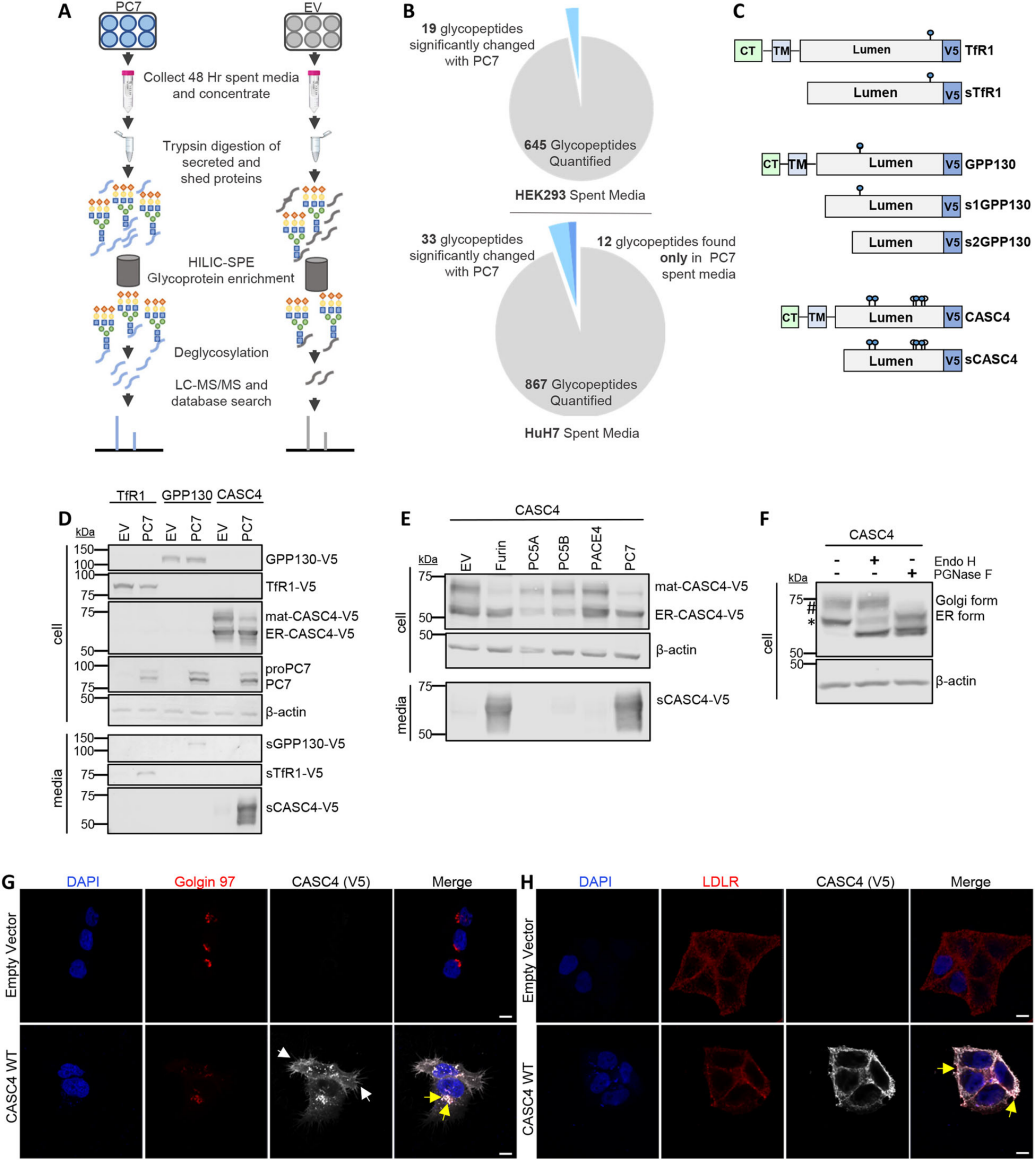
Mass spectrometry identifies two type-II transmembrane proteins shed by PC7 and Furin. **a** Schematic representation of the mass spectrometry strategy. The media from HEK293 or HuH7 cells overexpressing PC7 or empty vector were analyzed by LC-ESI-MS/MS and screened for quantitative changes in *N*-glycosylated soluble proteins. **b** Quantitative changes of 645 and 867 enriched glycosylated tryptic peptides from the spent media of HEK293 and HuH7 cells. **c** Schematic representation of human transferrin receptor 1 (TfR1), Golgi Phosphoprotein of 130 kDa (GPP130), and Cancer Susceptibility Candidate 4 (CASC4) identified in the analysis. Depicted are the cytosolic tail (CT), the transmembrane domain (TM), the luminal domain, and the C-terminal V5-tag. The blue circles are depicting potential *N*-glycosylation sites and the white circles are depicting potential *O*-glycosylation sites. **d** Western blot analysis of cell lysates and media from HEK293 cells expressing TfR1-V5, GPP130-V5, or CASC4-V5, with either empty pIRES-empty vector or hPC7. **e** Western blot analysis of cell lysates and media from HEK293 cells expressing CASC4-V5 with all the basic aa PCs. **f** Western blot analysis of cell lysates from HEK293 expressing CASC4-V5 treated with endo H or PGNase F. **g** Immunofluorescence analysis of permeabilized HeLa cells overexpressing CASC4-V5 colocalizing (yellow arrows) with Golgin-97, or in non-permeabilized cells with LDLR (**h**). These results are representative of three independent experiments. Scale: 10 µm.

To identify novel PC7-specific substrates, we used a mass spectrometry approach that analyses affinity purified *N*-glycoproteins secreted in the media (Fig. 1a). Analysis of secreted *N*-glycosylated products by this procedure avoids the limitations of the low concentrations of proteins of interest, high abundance of non-glycosylated plasma proteins in the incubation medium, or contamination by cytosolic proteins released from broken cells^28^. Indeed, we selectively enriched samples for *N*-glycosylated peptides using hydrophilic interaction chromatography solid phase micro-extraction (HILIC-SPE), before analyzing them by HPLC-ESI-MSMS (Fig. 1a). Accordingly, we compared the quantitative changes of 645 and 867 enriched glycosylated tryptic peptides from the spent media of human embryonic kidney cells (HEK293) and human hepatic (HuH7) cells, both endogenously expressing TfR1, overexpressing either human PC7 or an empty vector (EV) control, respectively (Fig. 1b, and raw data in Supplementary Tables S2 and S3). From HEK293 spent media with PC7, 19 glycopeptides had significantly enhanced levels, 18 of which exhibited potential PC-cleavage sites (K/R)-(2X_*n*_)-(K/R)↓ (Supplementary Table S2)^1^. From the HuH7 spent media with PC7, 33 glycopeptides had significantly enhanced levels and an additional 12 glycopeptides were only observed with PC7 expression (Supplementary Table S3). Of these, only ten parent glycoproteins exhibited potential PC-cleavage sites. These results allowed us to confirm previously known PC7 substrates, such as ADAM17^29^, Sortilin^8^, and human TfR1^9^, as well the identification of two novel substrates, the type-II transmembrane proteins CASC4 and GPP130/GOLIM4 (Supplementary Tables S2, S3 and Fig. 1c).

In order to confirm that CASC4 and GPP130 were cleaved by PC7, we co-expressed in HEK293 cells cDNAs coding for V5-tagged human TfR1, CASC4 or GPP130 with those encoding human PC7 or an empty vector control (Fig. 1d). Compared to control conditions, western blot (WB) analyses revealed the presence of secreted products (sCASC4 and sGPP130, or as control sTFR1) from cells co-expressing PC7, confirming that the membrane-bound CASC4 and GPP130 are shed by PC7, similarly to TfR1. In view of the connection of CASC4 to cancer (see later), in this study, we concentrated on the consequences of the shedding and functional modulation of CASC4 by PCs in details.

### CASC4 is shed by PC7 and Furin

To assess whether CASC4 shedding is specific to PC7, in HEK293 cells we co-expressed CASC4 with all the basic amino acid specific PCs (Furin, PC5A, PC5B, PACE4, and PC7)^1^, or with an empty vector control. WB analysis revealed the presence of a fragment released only in the media of cells expressing PC7 or Furin, but not from cells overexpressing the other PCs (Fig. 1e). Interestingly, the molecular sizes of the PC7 and Furin-shed products were similar (Fig. 1e), suggesting that they cleave CASC4 at, or close to, the same site. To further characterize the difference between the observed ~66 and ~75 kDa CASC4 cellular proteins (Fig. 1d, e), we treated the cell lysates with endoglycosidase H (endo H) that cleaves immature, mannose-rich sugars, or with PGNase F, which cleaves both immature and mature N-linked oligosaccharides (Fig. 1f). The data demonstrated that the ~66 kDa CASC4 is likely to reside in the endoplasmic reticulum (ER), since its apparent molecular size is reduced to ~61 kDa (*; ER-CASC4) when treated with endo H. We also noticed the presence of a residual amount of ~66 kDa CASC4 after endo H digestion, suggesting the presence of an *O*-glycosylated form that is not *N*-glycosylated. Indeed, the mature ~75 kDa CASC4 protein (#; mat-CASC4) is insensitive to endo H, suggesting it has exited the ER, but its size is reduced to both ~61 and ~66 kDa upon PGNase F digestion. As expected from the zymogen activation of Furin in the *trans*-Golgi network (TGN)^11,30^ and that of PC7 occurring in early endosomes^9,12^, only the ~75 kDa mat-CASC4 is processed by either PC7 or Furin (Fig. 1d, e). In support of these data, we compared the migration pattern of CASC4 expressed in CHO-K1 cells to that in CHO-ldlD cells, which are deficient in UDP-*N*-acetylgalactosamine and hence lack *O*-glycosylation activity^31^. The data clearly show that *O*-glycosylation adds ~11 kDa to the apparent molecular size of mature CASC4 (Supplementary Fig. S1).

Immunofluorescence staining of HeLa cells revealed that CASC4 co-localizes with the TGN marker Golgin-97 (yellow arrows, Fig. 1g). When cells were stained under non-permeabilized conditions, CASC4 is only detected at the cell surface, colocalizing with the cell-surface marker low-density-lipoprotein-receptor (LDLR) (yellow arrows, Fig. 1h). Interestingly, we observed a change in cell morphology upon CASC4 expression, providing a clue to elucidate a novel biological function of CASC4.

### CASC4 cleavage by PC7 and Furin occurs at Arg66↓

**Fig. 2:**
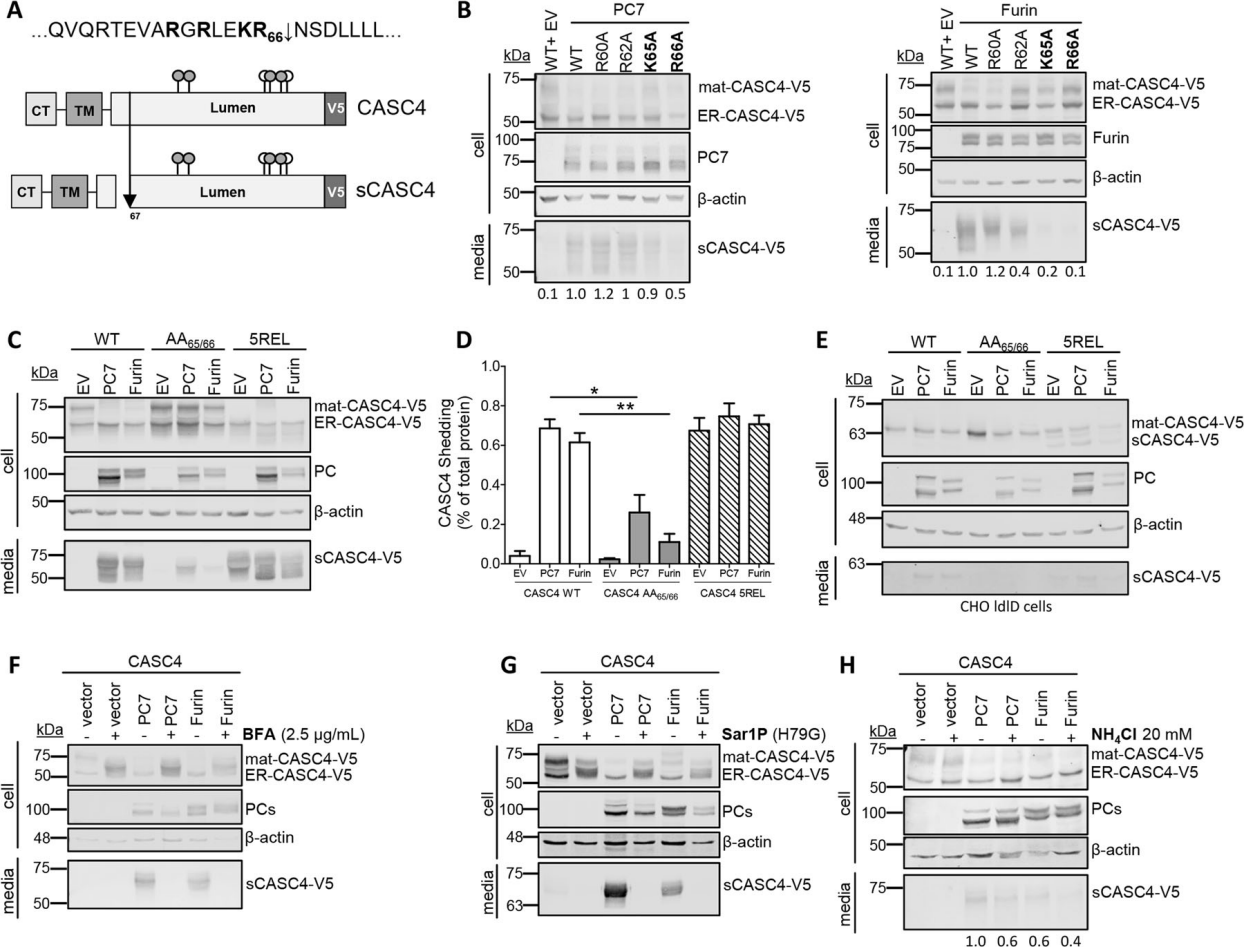
CASC4 cleavage by PC7 and Furin occurs after Arg66↓ in acidic compartment. **a** Schematic representation of the predicted cleavage site KR_66_↓ generating a N-terminal fragment and a luminal domain. The gray circles are depicting potential *N*-glycosylation sites and the white circles are depicting *O*-glycosylation sites. **b** Western blot analysis of cell lysates and media from HEK293 cells overexpressing CASC4-V5 WT and different point mutation (R60A, R62A, K65A, or R66A) co-expressed with either pIRES-empty vector or with hPC7 or hFurin. **c**, **d** Western blot analysis and quantifications of cell lysates and media from HEK293 cells overexpressing CASC4-WT-V5, CASC4-5REL-V5 optimally cleaved mutant, or a PC-non-cleavable AA_65/66_ site, co-expressed with either pIRES-empty vector or with hPC7 or hFurin. **e** Western blot analysis of cell lysates and media from CHO-ldlD cells overexpressing CASC4-V5 WT, CASC4-5REL optimally cleaved mutant or a PC-non-cleavable AA_65/66_ site, co-expressed with either pIRES-empty vector or with hPC7 or hFurin. **f** Western blot analysis of cells lysates and media from HEK293 cells overexpressing CASC4-V5 WT, pIRES-empty vector, and hPC7 or hFurin treated with Brefeldin A (2.5 µg/ml) or NH_4_Cl (20 mM) (**g**). **h** Western blot analysis of cell lysates and media from HEK293 cells overexpressing CASC4-V5 WT, pIRES-empty vector, dominant-negative Sar1P-(H79G), and/or with hPC7 or hFurin. These results are representative of three independent experiments. Error bars indicate averaged values ± standard error from the mean (SEM). *P*-values: *P ≤ 0.05, **P < 0.01, (two-sided Student’s *t-*test).

Because PC7 and Furin cleave substrates after single or paired basic amino acids (aa)^1^, we mutated the basic aa in the proposed P1-P2^31^ dibasic motif **KR**_66_**↓**, as well as the putative P5 **R**_60_ and P7 **R**_62_ sites into alanine (Fig. 2a, b). WB analyses of HEK293 cells co-expressing PC7 or Furin with WT-CASC4 or its Ala-mutants R60A, R62A, K65A or R66A, demonstrated that the K65A and especially R66A variants are resistant to shedding by PC7 and Furin (Fig. 2b), while the other mutants did not significantly affect shedding. To further emphasize that **KR**_**66**_**↓** is the shedding site, we generated CASC4 mutant proteins harboring an optimized PC-site RRRRR_**66**_EL^1,32^ (called 5REL), or a PC-non-cleavable AA_65/66_ mutant. Accordingly, in HEK293 cells, processing of the CASC4-AA_65/66_ mutant protein was impaired compared to WT (Fig. 2c, d). Expression of the 5REL mutant generated a soluble fragment, even when overexpressed with an empty vector control (Fig. 2c, d), with an apparent molecular size similar to that of the PC7/Furin-cleaved form of WT-CASC4, supporting that **KR**_66_**↓** is the shedding site. To demonstrate that the heterogeneity of the secreted forms of CASC4 is due to *O*-glycosylation (Fig. 2c), we co-expressed CASC4-WT and its mutants 5REL and AA_65/66_ in CHO-ldlD cells that cannot *O*-glycosylate proteins^33^. As expected, the secreted sCASC4 now migrates as a sharp protein band (~61 kDa), confirming the *O*-glycosylation of CASC4 (Fig. 2e). Taken together, these data confirm that CASC4 cleavage occurs at **KR**_66_**↓**NS, which generates a secreted luminal domain and a short N-terminal domain (NTD) composed of the cytosolic tail and transmembrane domain.

### Shedding occurs in acidic compartments of the secretory pathway

Brefeldin A abrogated CASC4 shedding by PC7 and Furin (Fig. 2f). Since Brefeldin A blocks transport of secretory proteins out from the ER^34^, this suggests that shedding occurs in a post-ER compartment. The same conclusion was reached upon expression of the dominant-negative Sar1p-(H79G) (Fig. 2g), a GTP-restricted mutant that blocks COP-II vesicle formation^35^. Hence, proper COP-II vesicle formation and trafficking from the ER to the Golgi is necessary for PC7 and Furin to shed CASC4. In addition, we used the cell non-permeable general PC inhibitor D6R, which inhibits PC activity at the cell surface, in comparison to decanoyl-RVKR-chloromethylketone, a cell-permeable PC inhibitor^9^. Our results show that CASC4 cleavage by both PC7 and Furin is reduced by dec-RVKR-CMK, suggesting that the cleavage occurs intracellularly (Supplementary Fig. S2). On the other hand, both D6R and Dynasore (an inhibitor of endocytic vesicles^9^) treatments do not inhibit CASC4 shedding, strongly suggesting that shedding occurs intracellularly and does not require endocytosis from the plasma membrane (Supplementary Fig. S2). Taken together these results combined with our observations of a reduced shedding upon treatment of cells with the alkalinizing agent ammonium chloride (NH_4_Cl) to block the acidification of intracellular compartments (Fig. 2h), led us to conclude that shedding occurs in an acidic compartment such as TGN or endosomes originating from the TGN, where enzymatic activity was previously described for both PC7 and Furin^9,12,31^.

### CASC4 is expressed in metastatic breast cancer cells and its association with PC7 predicts poor prognosis in breast cancer patients

**Fig. 3:**
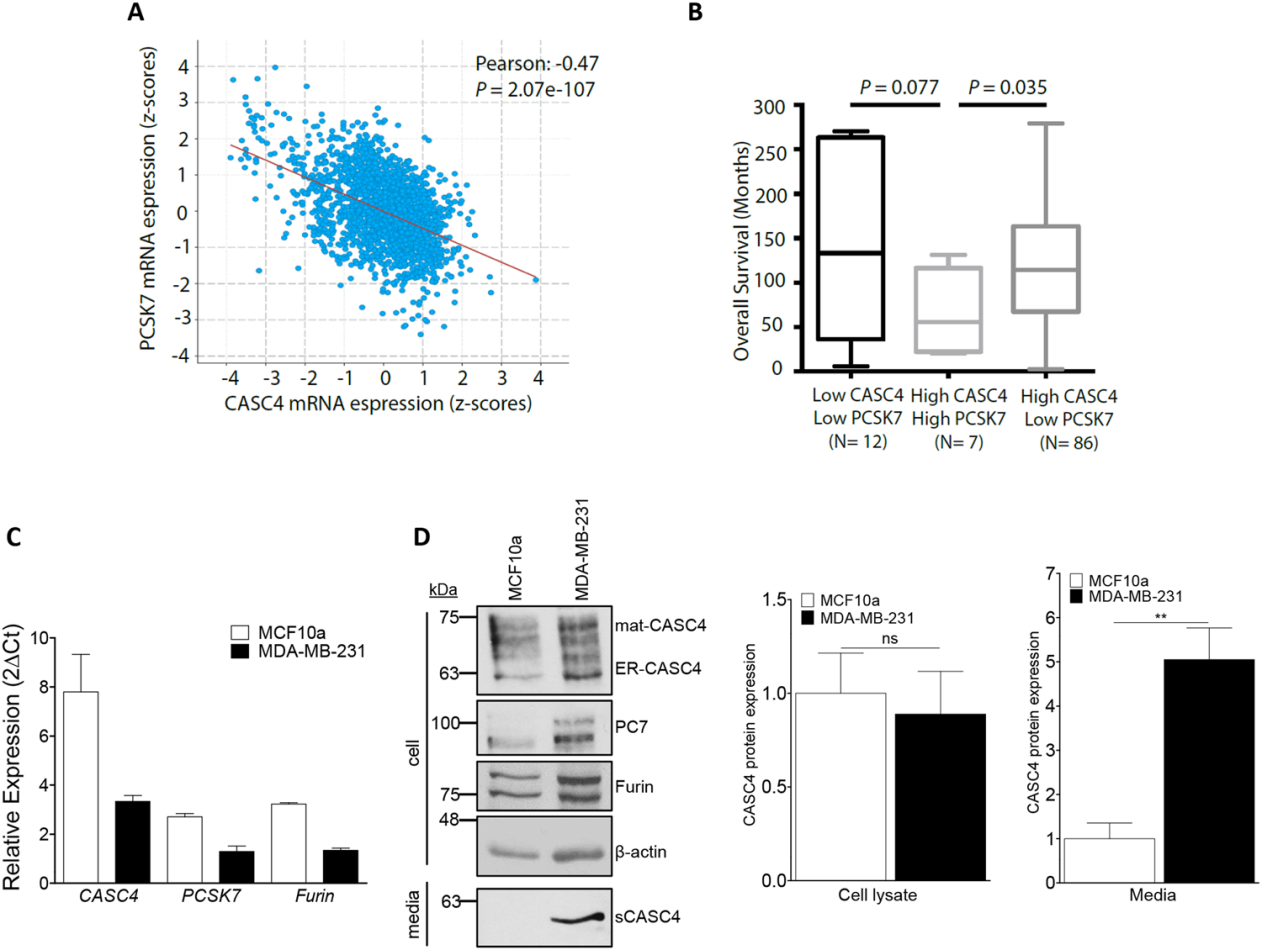
CASC4 association with PC7 predicts poor prognosis in breast cancer patients. **a** Correlation of *CASC4* and *PCSK7* mRNA expression levels (in *z*-scores) in the METABRIC patients’ data set. **b** Association of the expression levels of CASC4 and PCSK7 genes with the survival patients’ rate. **c** qPCR analysis of *hCASC4*, *hPCSK7*, and *hFurin* in MCF10a and MDA-MB-231 cells. **d** Western blot analysis and quantifications of cell lysates from MCF10a or MDA-MB-231 cells. *P*-values: **P < 0.01, n.s. not significant (two-sided Student’s *t*-test). All experiments were repeated at last three times and center values represent the mean ± standard error from the mean.

CASC4 is an uncharacterized protein reported to be associated with a potentially bad prognosis in breast cancer^13^, and its gene is aberrantly spliced in breast cancer cells^15^. In addition, metastatic MDA-MB-453 triple-negative breast cancer cells exhibit increased levels of secreted sCASC4 compared to non-cancerous breast cell lines^17^. This motivated us to investigate the potential biological role of the PC7-CASC4 association in the context of breast cancer. First, we interrogated the METABRIC clinical data set, the largest breast cancer data set encompassing genomic data from ~2500 samples. Although, *CASC4* mRNA levels showed a significant negative correlation with those of *PCSK7* (Fig. 3a), the expression levels of the latter could differentiate the survival rate of the patients with high CASC4 levels (Fig. 3b). Indeed, patients with high *CASC4* and high *PCSK7* expression levels had a significantly worse prognosis that those with high *CASC4* but low *PCSK7* mRNA levels (Fig. 3b).

Second, we analyzed the expression levels of *CASC4*, *PCSK7*, and *Furin* mRNA levels in MCF10a (human non-cancerous breast epithelial cell line) versus MDA-MB-231 (highly metastatic triple-negative breast cancer cell line) cells by qPCR. The three genes are ~2–2.5-fold more expressed in MCF10a versus MDA-MB-231 (Fig. 3c). However, at the protein level, WB analysis revealed that while CASC4 is similarly expressed in both cell lines, its shedding into the media was mostly observed in MDA-MB-231 cells (Fig. 3d). In addition, the protein expression levels of PC7 and Furin were similar in both cells (Fig. 3d). We suggest that the TGN localization of CASC4 (Fig. 1g) and that of the active form of its processing enzymes^12^ may provide a favorable environment to allow its shedding in MDA-MB-231 cells compared to MCF10a cells. In addition, we also assessed by qPCR the expression levels in these cells of the endogenous inhibitors of Furin and PC7, namely PAR1^36^, GBP2, and GBP5^37^. The data show that *PAR1* is expressed at ~6-fold higher levels in MCF10a than in MDA-MB-231 cells, whereas *GBP2* is unchanged in both cell lines and *GBP5* is barely expressed (Supplementary Fig. S3). Thus, the low expression of the Furin/PC7 inhibitor PAR1^36^ in MDA-MB-231 cells rationalizes the higher activity of Furin and PC7 therein. Altogether these data suggest that the PC7-CASC4 association and specifically the PC7-mediated shedding of CASC4 might have functional consequences in terms of breast cancer aggressiveness (i.e., metastasis).

### CASC4 modulates cell migration and invasion

**Fig. 4:**
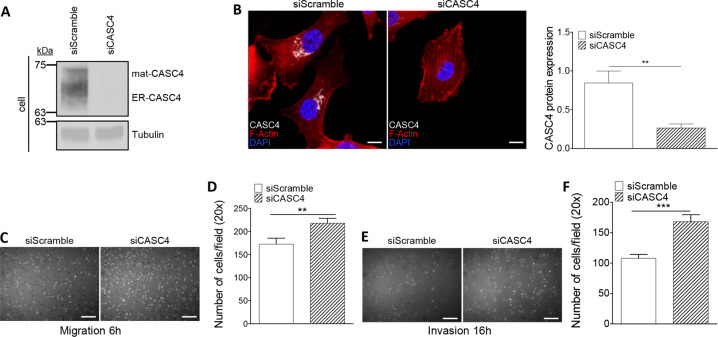
CASC4 knockdown increases migration and invasion in MDA-MB-231 metastatic breast cancer cells. **a** Western blot analysis and quantification of cell lysates from MDA-MB-231 cells after 48 h siRNA knockdown of endogenous CASC4. **b** Immunofluorescence analysis of MDA-MB-231 cells after 48 h siRNA knockdown of endogenous CASC4 stained for CASC4 (white labeling), phalloidin (red labeling) and nucleus stained with DAPI (blue labeling). The ability of MDA-MB-231 cells, after 48 h siRNA knockdown of endogenous CASC4 to migrate (6 h) (**c**, **d**) or invade (16 h) (**e**, **f**) was assessed by counting the number of cells stained with DAPI on the underside of a polycarbonate membrane under a phase contrast microscope (×20). These results are representative of at least three independent experiments. Error bars indicate averaged values ± standard error from the mean (SEM). *P*-values: **P < 0.01, ***P ≤ 0.001 (two-sided Student’s *t-*test). Scale: 10 µm.

The cytoskeletal extensions observed upon overexpression of CASC4 in Hela cells (Fig. 1g), and the correlation between *PCSK7-CASC4* mRNA levels with breast cancer patients’ survival (Fig. 3a, b) suggested that CASC4 may impact cell migration and invasion, two essential steps of the metastatic process. We thus investigated whether siRNA knockdown of CASC4 alters cell migration and invasion in MDA-MB-231 cells. The siRNA-induced silencing was efficient since it reduced by ≥80% endogenous CASC4 protein, as observed by WB (Fig. 4a) and immunofluorescence (Fig. 4b). Depletion of CASC4 resulted in a significant increase in cell migration (+30%) and invasion (+60%) as assessed by Boyden Migration and Invasion Assays, respectively (Fig. 4c–f). These results suggest that expression of CASC4 in MDA-MB-231 cells reduces their cell migration and invasion potential.

**Fig. 5:**
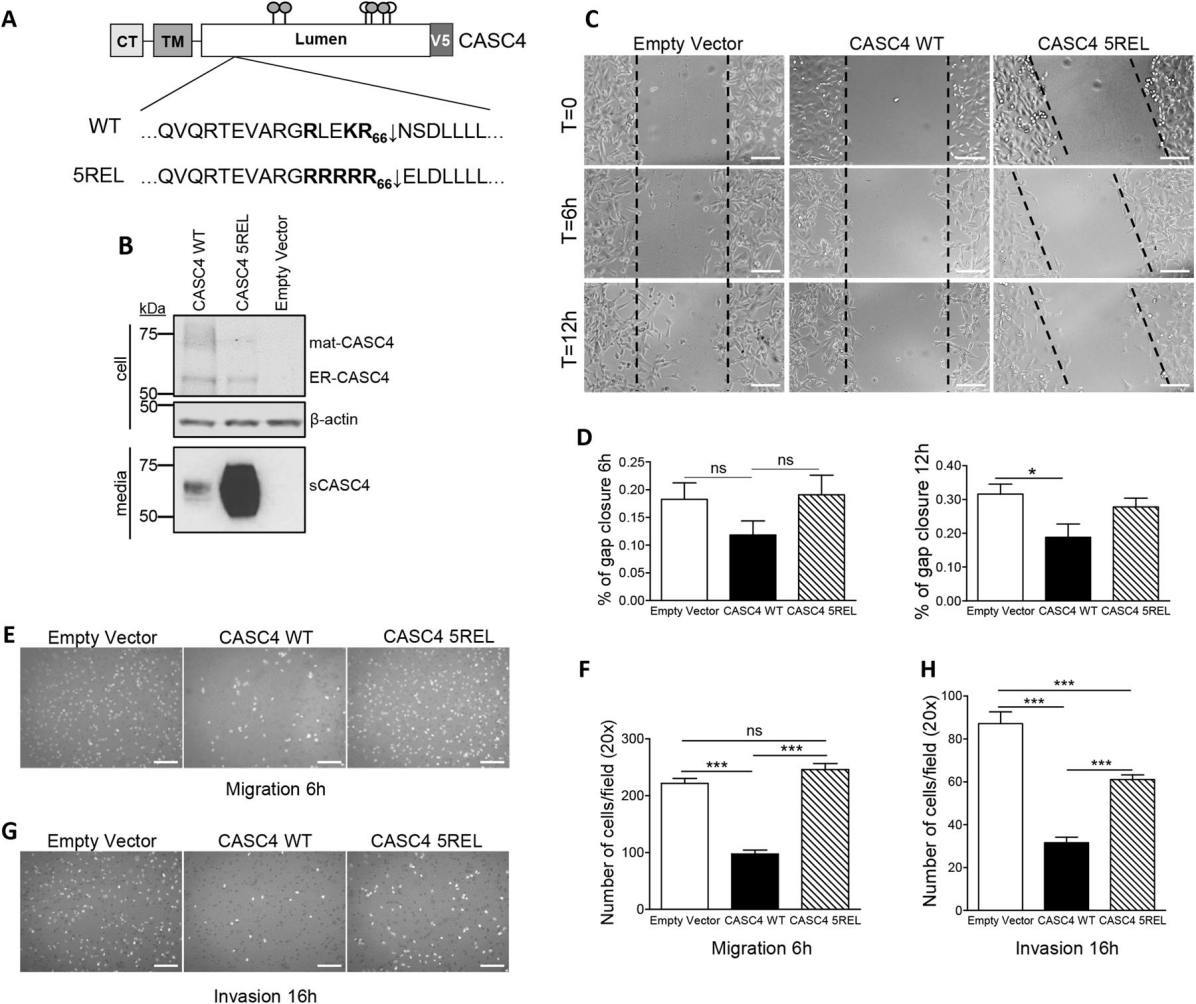
CASC4 overexpression decreases cell migration and invasion. **a** Schematic representation of CASC4-WT and amino acid mutations used for the generation MDA-MB-231 stable cells. **b** Western blot analysis of lysates from MDA-MB-231 stable cells expressing pIRES-empty vector, CASC4-WT-V5, or CASC4-5REL-V5. **c**, **d** Wound healing assay images and quantifications after 6 h, 12 h post-cell monolayer scratching in MDA-MB-231 stable cell lines. **e**–**h** The ability of MDA-MB-231 stable cells to migrate (6 h) (**e**–**f**) or invade (16 h) (**g**–**h**) was assessed by counting the number of cells stained with DAPI on the underside of a polycarbonate membrane under a phase contrast microscope (×20). These results are representative of at least three independent experiments. Error bars indicate averaged values ± standard error from the mean (SEM). *P*-values: *P ≤ 0.05, ***P ≤ 0.001, n.s. not significant (two-sided Student’s *t-*test).

Accordingly, we generated MDA-MB-231 cells stably expressing CASC4-WT, CASC4-5REL (with the constitutively PC-cleaved RRRRR_66_**↓**EL motif), or an empty vector control (Fig. 5a). We confirmed the protein expression in these cells by WB analysis (Fig. 5b). Since the lack of CASC4 expression resulted in enhanced migration and invasion (Fig. 4c–f), we evaluated the migration potential of the stable cell lines first by using a wound healing assay. Compared to control or cells expressing CASC4-5REL, only expression of CASC4-WT resulted in significant inhibition of wound closure post-scratching, especially evident after 12 h (Fig. 5c, d). In complementary Boyden Migration and Invasion Assays (Fig. 5e–h), CASC4-WT overexpression significantly reduced cell migration (−50%) and invasion (−70%), supporting a protective role of CASC4 as a negative regulator of cellular movement. Although overexpression of CASC4-5REL did not show a significant migration phenotype (Fig. 5e, f), its effect on invasion was intermediate between WT and control (Fig. 5g, h). Taken together, these data suggest that CASC4 represses cellular migration, possibly by acting on key players orchestrating the cellular architecture, and that CASC4 shedding by PCs largely prevents this effect.

### CASC4 enhances the number of FA formation and impairs Cdc42 activation

**Fig. 6:**
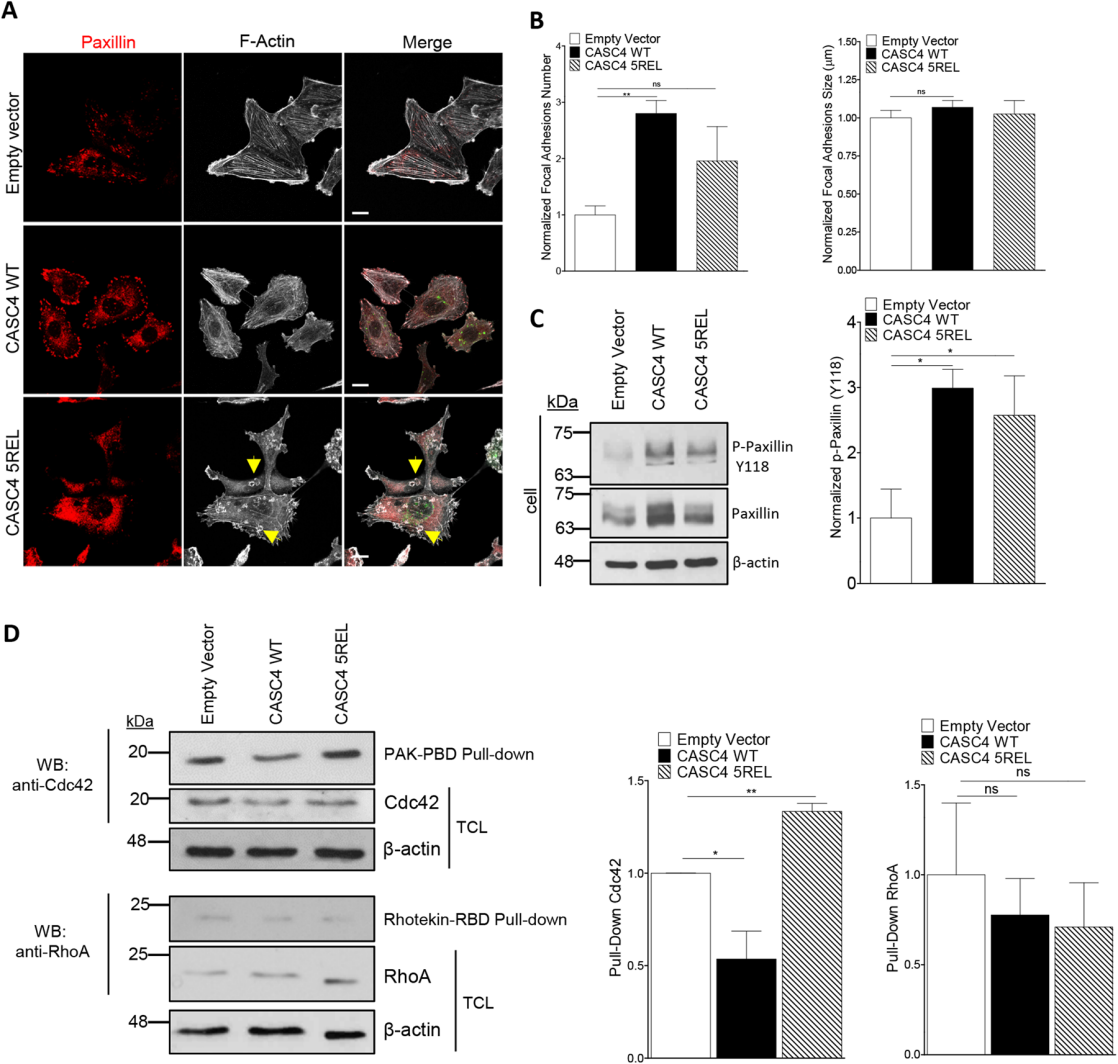
CASC4 enhances focal adhesions and perturb actin architecture. **a** Immunofluorescence analysis of stable MDA-MB-231 cell lines expressing pIRES-empty vector, CASC4-WT or CASC4-5REL stained for focal adhesion marker (Paxillin; red labeling), phalloidin (F-actin; white labeling), and V5 (green labeling). Yellow arrows are highlighting round actin circles generated in cells expressing CASC4-5REL. **b** Quantifications of focal adhesions (Paxillin-positive areas) number and size. **c** Western blot analysis and quantifications of cell lysates from MDA-MB-231 stable cells showing endogenous paxillin and p-paxillin (Y118). **d** Western blot analysis and quantifications of cells lysates from MDA-MB-231 stable cells incubated with the indicated GST-fusion proteins bound to glutathione beads. The precipitated proteins were detected by immunoblotting with anti- Cdc42 or anti-RhoA antibodies. TCL total cell lysate. These results are representative of at least three independent experiments. Error bars indicate averaged values ± standard error from the mean (SEM). *P*-values: *P ≤ 0.05 **P < 0.01, n.s. not significant (two-sided Student’s *t-*test). Scale: 10 µm.

To investigate how CASC4 interferes with cellular migration/invasion, we analyzed the actin architecture and FAs by immunofluorescence for phalloidin- and paxillin staining, respectively. Interestingly, CASC4-WT cells exhibited a higher number and a trend for larger paxillin-positive FA complexes (Fig. 6a, b), as well as ~3-fold more Tyr_118_-phosphorylated paxillin (Fig. 6c). In addition, the overall architecture of CASC4-WT cells was severely impaired, as evidenced by the induction of actin stress fibers at the expense of cortical actin (Fig. 6a). This architectural cellular phenotype could explain the observed reduced migration observed in CASC4-WT cells (Fig. 5c–h). The actin architecture in cells expressing CASC4-5REL, while also severely disrupted, exhibited a very different phenotype with a completely disorganized paxillin staining (Fig. 6a).

We next characterized in our stable cell lines the activation/inactivation of Rho GTPases, which are molecular switches that control actin cytoskeleton and FA dynamics^38,39^. The levels of the active forms of the Rho GTPases Cdc42 and RhoA implicated in FA turnover^40,41^ were quantified by specific GST pull-downs. The data revealed a significant decrease in the levels of active Cdc42 in cells overexpressing CASC4-WT, compared to empty vector and CASC4-5REL, whereas those of active RhoA remained unchanged (Fig. 6d). This suggests that CASC4-WT, but not CASC4-5REL, blunts the activation of Cdc42, resulting in increased assembly of FA and stress fibers (Fig. 6a).

### CASC4 NTD impairs actin organization and induces invadopodia-like structures

**Fig. 7:**
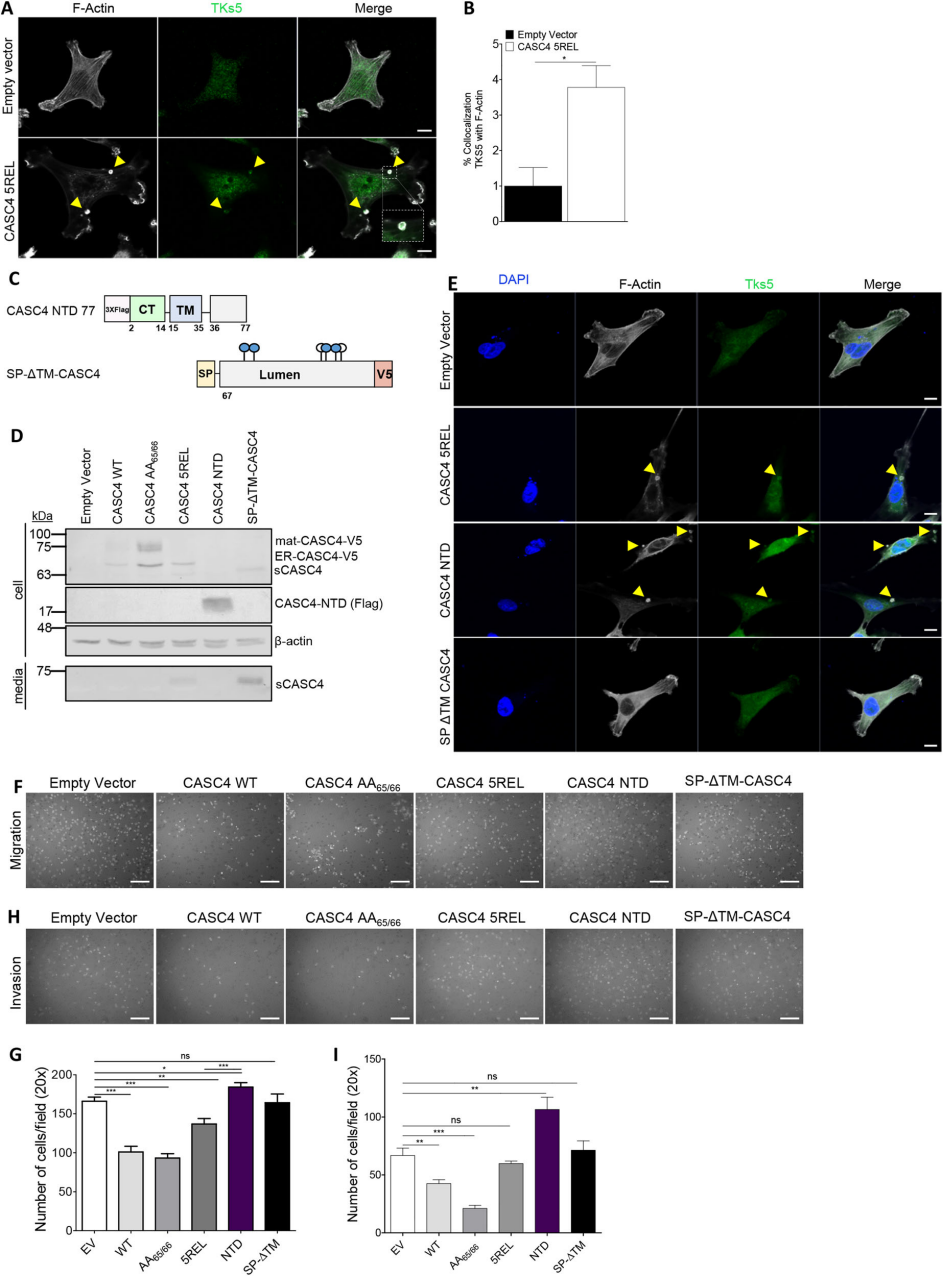
CASC4 N-terminal domain (NTD) induces podosome-like structures. **a**, **b** Immunofluorescence analysis and quantification of MDA-MB-231 stable cells stained for phalloidin (F-actin; white labeling) or tyrosine kinase substrate 5 (TKS5; green labeling). **c** Schematic representation of CASC4 truncated mutant (NTD) with a 3xflag in N-terminal and SP-ΔTM-CASC4 with a V5 in C-terminal. The blue circles are depicting potential *N*-glycosylation sites and the white circles are depicting *O*-glycosylation sites. **d** Western blot analysis of cell lysates and media from MDA-MB-231 cells transiently transfected with different CASC4 constructs. **e** Immunofluorescence analysis of transiently transfected MDA-MB-231 cells stained for phalloidin (F-actin; white labeling), tyrosine kinase substrate 5 TKS5 (green labeling), and nucleus stained with DAPI (blue labeling). Yellow arrows represent colocalization between F-actin and TKS5. The ability of transiently transfected MDA-MB-231 cells to migrate (6 h) (**e**, **f**) or invade (16 h) (**g**, **h**) was assessed by counting the number of cells stained with DAPI on the underside of a polycarbonate membrane under a phase contrast microscope (×20).These results are representative of at least three independent experiments. Error bars indicate averaged values ± standard error from the mean (SEM). *P*-values: *P ≤ 0.05, **P < 0.01, ***P ≤ 0.001 n.s. not significant (two-sided Student’s *t*-test). Scale: 10 µm.

Notably, cells expressing CASC4-5REL presented round actin rings (depicted with yellow arrows) (Fig. 6a). These structures are reminiscent of circular dorsal ruffles, actin structures present on the dorsal surface of cells in response to stimuli (i.e., EGF, PDGF)^42^, and/or invadosomes (invadopodias/podosomes) structures implicated in actin remodeling and invasion in both cancerous (invadopodias) and non-cancerous cells (podosomes)^18,19^. Since colocalization of F-actin with protein tyrosine kinase substrate 5 (Tks5) and extracellular matrix degradation can define invadosomes^19^, we characterized these structures by immunofluorescence staining for F-actin and Tks5. The data showed that Tks5 staining co-localizes (yellow arrows) with the F-actin structures in CASC4-5REL cells (Fig. 7a, b), suggesting that these actin structures were invadopodias/podosomes. We next investigated whether the NTD or the C-terminal luminal fragment generated by PC7/Furin is/are implicated in invadopodias induction. Thus, we generated two constructs, one with a stop codon at 11-residues after the shedding site (aa 1–77, likely processed at Arg_66_↓), to create an artificial NTD fragment, and another secretory protein mimicking the luminal shed domain with an N-terminal PCSK9 signal peptide (SP)^43^ fused to Asn_67_ following the shedding site at Arg_66_ (SP-ΔTM) (Fig. 7c). We confirmed the expression of these constructs in MDA-MB-231 cells by WB analyses (Fig. 7d) and immunocytochemistry (Supplementary Fig. S4). We next assessed the presence of the actin rings structures in cells expressing the different constructs by immunofluorescence (Fig. 7e). Cells expressing the 5REL and especially the membrane-bound NTD constructs exhibit the presence of round actin structures colocalizing with Tks5 (yellow arrows). To test the NTD role in inducing invadopodias formation, likely influencing migration/invasion, we transiently expressed all constructs in MDA-MB-231 cells and performed Boyden Invasion and Migration Assays. First, we showed that the double Ala-mutant of the shedding site (CASC4-AA_65/66_, Fig. 2c) is ~2-fold more active in reducing invasion than the WT-CASC4 (Fig. 7h, i), supporting the protective role of full-length CASC4. In contrast, expression of the NTD significantly enhanced migration/invasion, whereas expression of the SP-ΔTM mutant had no effect (Fig. 7f–i). Thus, the N-terminal fragment generated upon shedding of CASC4 is mainly responsible for the cytoskeletal disruption observed in the CASC4-5REL cells.

## Discussion

The PCs have key roles in both health and disease states by cleavage of precursor proteins^1^, which results in the bioactivation of proteins, but sometimes may also generate cleaved products endowed with novel functions^44,45^. While the roles of Furin in proliferative and infectious diseases have been extensively characterized^10,46–49^, those of PC7 are barely defined^8,9,12^. One of the specific functions of PC7 is the shedding of the human type-II TfR1^9^, resulting in the secretion of a circulating sTfR1 that correlates with iron deficiency^50^. These data and those of the present study suggest that PC7 may shed several type-II transmembrane proteins, and would not represent a rare phenomenon, but rather provide a mechanism to modulate their functions and possibly generate new ones.

A proteomic screen from the media of HuH7 cells identified three shed type-II transmembrane proteins, TfR1, as well as CASC4 and GPP130. We demonstrated that only PC7 and Furin can shed CASC4 at Arg_66_↓ and GPP130 (not shown) in post-ER acidic compartments. This CASC4 cleavage generates an N-terminal membrane-bound fragment (NTD; aa 2–65) and a secreted C-terminal fragment starting at Asn_67_. The only available information on CASC4 is its association with HER2^**+**^ overexpression and its differential splicing in breast cancer^13,15^ and glioblastoma^16^. Our METABRIC data indicated that patients with high *PCSK7* and high *CASC4* had significantly worse prognosis than those with high *CASC4* but low *PCSK7*. In addition, we demonstrated that endogenous shedding of CASC4 is only observed in triple-negative breast cancer cells MDA-MB-231. We thus hypothesized that CASC4 and its shedding are relevant in breast cancer aggressiveness/metastasis. Initial evidence revealed that knockdown of *CASC4* mRNA significantly increased cell migration and invasion, suggesting a protective role of CASC4.

**Fig. 8:**
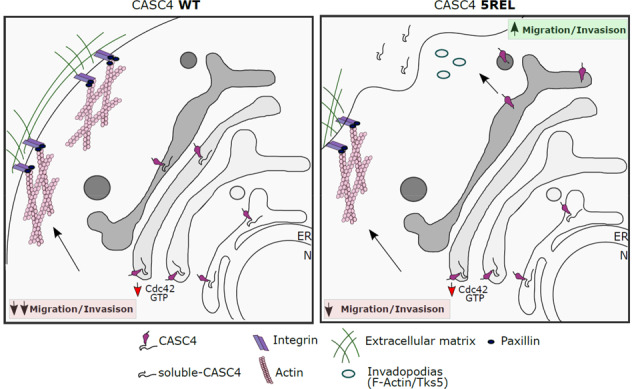
Schematic representation of CASC4 biological functions along the secretory pathway. Depicted are full-length CASC4-WT in the early secretory pathway interfering with the Rho GTPase Cdc42-GTP activation. The decrease in Cdc42 activation results in increased paxillin-positive staining focal adhesions which slows down migration (left panel). The dual roles for CASC4-5REL functions are depicted as the protein remain full-length (CASC4-WT) in the early secretory pathway, but is shed trafficking along the secretory pathway which generates membrane-bound N-terminal domain (NTD), which induces the formation of podosome-like structures (right panel) and increases migration, which results in an intermediate migratory phenotype.

To explore the functional consequences of CASC4 shedding, we generated three cells lines expressing either a control empty vector, CASC4-WT or CASC4-5REL. The data revealed that overexpressed CASC4 enhanced the number of FAs, in part by blunting the activation of the Rho GTPase Cdc42, supporting its effect on the reduced migratory potential of these cells. It is not surprising that Cdc42 activity could be modulated by Golgi-localized CASC4 since Cdc42 localizes to the Golgi apparatus and interacts with regulators of cytoskeleton remodeling and centrosome organization^51^. We further demonstrated that shedding of CASC4 into sCASC4 is a critical event that transforms the protective function of CASC4-WT into a pathogenic one due to the generation of the NTD (Fig. 8). Since Furin/PC7 shedding likely occurs in the TGN and/or endosomes, CASC4-5REL would remain as full length until it reaches these intracellular destinations, and would partially reduce the levels of active Cdc42 in early Golgi compartments (Fig. 8), like CASC4-WT. This may rationalize the intermediate invasion phenotype observed in cells expressing CASC4-5REL, which when shed later along the secretory pathway would generate an NTD that rather increases podosome formation, known to be associated with enhanced invasion^18,19^. More detailed studies are needed to define the underlying mechanism behind the activity of the NTD in enhancing the formation of podosome-like structures. Since we do not know the physiological function of CASC4, future work will also be needed to investigate the function of the luminal domain of this protein. Similar to other members of the GOLM1 family, CASC4 may act as a co-receptor, as reported for GOLM1/GP73 for the EGF receptor^52^.

The fact that experimental expression of different forms of CASC4 (WT, AA_65/66_ or 5REL) leads to different/opposing biological consequences in the context of breast cancer, suggests that this protein might have dual actions depending on the associated activity of PC7/Furin. Breast cancer can be categorized pathologically into three main categories: Estrogen/progesterone receptor (ER/PR)-positive, HER2^**+**^, or Triple-Negative Breast Cancer (TNBC). Interestingly, in two different clinical breast cancer data sets, METABRIC and TCGA, we found that *CASC4* expression positively correlates with the ER/PR status (Supplementary Fig. S5). Correlating the *CASC4* mRNA levels to the different molecular subtypes of breast cancer by the PAM50 classification showed similarly that luminal tumors, that are usually ER/PR-positive and have best prognosis, express the highest levels of *CASC4* (Supplementary Fig. S5). These evidences strongly pinpoint the significance of exploring the dual roles of CASC4 and its PC7/Furin-shed form in order to better understand the role of CASC4 in different breast cancer subtypes.

Interestingly, analysis of the genes implicated in cancer on chromosome 11, revealed that the locus 11q23.3 is associated with a high incidence of ovarian, breast and uterine cancer (human cancer proteome database http://canprovar2.zhang-lab.org/chr/chr11.php). Coincidentally, this is the locus of *PCSK7* that resides close to the gene-cluster *APOA5/APOA4/APOC3/APOA1*, a region implicated in the regulation of lipoprotein metabolism (Supplementary Fig. S6)^53^.

In conclusion, our results provide a framework for deciphering the biological functions of CASC4 and more importantly suggest that inhibitors of Furin/PC7 may find clinical applications in breast and ovarian cancers^1,5,48^.

## Supplementary information

Supplementary Legends

Supplementary Table S1

Supplementary Table S2

Supplementary Table S3

Supplementary Figure S1

Supplementary Figure S2

Supplementary Figure S3

Supplementary Figure S4

Supplementary Figure S5

Supplementary Figure S6
