# Supplementary material for: Shedding of cancer susceptibility candidate 4 by the convertases PC7/furin unravels a novel secretory protein implicated in cancer progression
Source: Cell Death Dis. 2020 Aug 20;11(8):665. doi: 10.1038/s41419-020-02893-0 (PMC7441151; doi:10.1038/s41419-020-02893-0)
Supplement: Supplementary file 1 — Supplementary Legends [file 41419_2020_2893_MOESM1_ESM.docx]

**Supplementary Figure S1**

**CASC4 is N- and O-glycosylated.** A) Western blot analysis of cell lysates and media from CHO-K1 and CHO-ldlD cells overexpressing CASC4-V5 WT. Arrows represent the mature form of CASC4 (#; mat-CASC4) and its ER form (*; ER-CASC4). B-C) Western blot analysis of cell lysates from CHO-K1 and CHO-ldlD cells expressing CASC4-V5 treated with endo H or PGNase F. These results are representative of three independent experiments.

**Supplementary Figure S2**

**CASC4 is cleaved by PC7 and Furin intracellularly.** Western blot analysis of cell lysates from HEK293 cells expressing CASC4-V5 treated with decanoyl-RVKR-cmk, hexapeptide (D-Arg)_6_, or Dynasore. These results are representative of three independent experiments. Error bars indicate averaged values ± standard error of the mean (SEM). P values: *, p ≤ 0.05, ns: not significant (two-sided Student’s t-test).

**Supplementary Figure S3**

**Natural PC7 and Furin inhibitor PAR1 is 6X more expressed in MCF10a compared to MDA-MB-231 cells.** PAR1, GBP2 and GBP5 mRNA levels in MCF10a and in MDA-MB-231 cells. These results are representative of three independent experiments. Error bars indicate averaged values ± standard error of the mean (SEM). P values: **, p <0.01, ns: not significant (two-sided Student’s t-test).

**Supplementary Figure S4**

**CASC4-NTD and SP-ΔTM-CASC4 are well expressed in MDA-MB-231 cells.** Immunofluorescence analysis of transiently transfected MDA-MB-231 cells stained for phalloidin (F-actin; white labeling), CASC4 (V5 or Flag tags) (green labeling) and nucleus stained with DAPI (blue labeling). Scale: 10 µm.

**Supplementary Figure S5**

**METABRIC and TCGA patients’ raw data.** Raw data (with samples IDs) used to investigate the correlation between CASC4 mRNA levels in METABRIC and TCGA patients’ datasets. PR=Progesterone receptor, ER=Estrogen receptor.

**Supplementary Figure S6**

**Cancer locus of PCSK7.** Map of the chromosome 11 with different cancer incidence, where PC7 is highlighted in ovarian, breast and uterine cancer, and is located in close proximity with the apolipoprotein locus.

**Supplementary Table S1:**

**Oligonucleotides used in RT-quantitative PCR.**

**Supplementary Table S2**

**Glycopeptides significantly enriched from the spent media of HEK293 cells overexpressing human proprotein convertase 7.** NFEV: Glycopeptide not identified from HEK293 spent media transfected with empty vector as control and significantly different from media from PC7 expressing cells. Significance defined as p<0.05 by a two-sided Student’s *t-*Test.

**Supplementary Table S3:**

**Glycopeptides significantly enriched from the spent media of HuH7 cells overexpressing human proprotein convertase 7.** NFEV: Glycopeptide not identified from HuH7 spent media transfected with empty vector as control. NFEVS: Not found in EV and significant from PC7 spent media. Significance defined as p<0.05 by a two-sided Student’s *t*-Test.
