# Supplementary material for: Shedding of cancer susceptibility candidate 4 by the convertases PC7/furin unravels a novel secretory protein implicated in cancer progression
Source: Cell Death Dis. 2020 Aug 20;11(8):665. doi: 10.1038/s41419-020-02893-0 (PMC7441151; doi:10.1038/s41419-020-02893-0)
Supplement: Supplementary file 2 — Supplementary Table S1 [file 41419_2020_2893_MOESM2_ESM.docx]

| Gene | Forward | Reverse |
| --- | --- | --- |
| *CASC4* | GAAGAACCCTCAAGCAATCATATTC | CCTGCATCACCACCTCTTTTG |
| *PCSK7* | GGACCTACAGGCTTGTCATC | CCACTCATGGCACTCTCTAACA |
| *Furin* | CACCAGCGAAGCCAACAACT | TGCCATAGAGTACGAGGGTGAA |
