# Supplementary material for: Shedding of cancer susceptibility candidate 4 by the convertases PC7/furin unravels a novel secretory protein implicated in cancer progression
Source: Cell Death Dis. 2020 Aug 20;11(8):665. doi: 10.1038/s41419-020-02893-0 (PMC7441151; doi:10.1038/s41419-020-02893-0)
Supplement: Supplementary file 3 — Supplementary Table S2 [file 41419_2020_2893_MOESM3_ESM.docx]

| Protein ID | Gene ID | Protein Name | Ratio glycopeptide intensity PC7/EV | N-linked glycopeptide  identified |
| --- | --- | --- | --- | --- |
| P02786 | TFRC | Transferrin receptor protein 1 | 52.81106 | KDFEDLYTPVNGSIVIVR |
| P02786 | TFRC | Transferrin receptor protein 1 | 29.93287 | KQNNGAFNETLFR |
| Q13433 | SLC39A6 | Zinc transporter ZIP6 | 7.908147 | YGENNSLSVEGFR |
| Q13433 | SLC39A6 | Zinc transporter ZIP6 | 2.976814 | KTNESVSEPR |
| Q16549 | PCSK7 | Proprotein convertase subtilisin/kexin type 7 | NFEVS | CAGEIAAVPNNSFCAVGVAYGSR |
| Q16549 | PCSK7 | Proprotein convertase subtilisin/kexin type 7 | NFEVS | DINVTGVWERNVTGR |
| Q16549 | PCSK7 | Proprotein convertase subtilisin/kexin type 7 | NFEVS | DINVTGVWERNVTGR |
| P49641 | MAN2A2 | Alpha-mannosidase 2x | 12.52052 | NLGFNCTTSQGK |
| P55268 | LAMB2 | Laminin subunit beta-2 | 4.758911 | NTSAASTAQLVEATEELRR |
| A2RU67 | KIAA1467 | Uncharacterized protein KIAA1467 | 15.38932 | APDSNCSNLLITTR |
| Q96MM7 | HS6ST2 | Heparan-sulfate 6-O-sulfotransferase 2 | 3.47587 | YNFTRGDLLR |
| Q9UK76 | HN1 | Hematological and neurological expressed 1 protein | NFEVS | GVDPNSRNSSR |
| Q9UK76 | HN1 | Hematological and neurological expressed 1 protein | NFEVS | VLRPPGGGSNFSLGFDEPTEQPVRK |
| Q8WXG9 | GPR98 | G-protein coupled receptor 98 | 0.125277 | ISEENTTAR |
| P10253 | GAA | Lysosomal alpha-glucosidase | 3.670205 | NNTIVNELVR |
| O75976 | CPD | Carboxypeptidase D | 7.709885 | NNSNNFDLNR |
| Q9H8M5 | CNNM2 | Metal transporter CNNM2 | 11.78985 | VYGQNINNETWSR |
| Q6P4E1 | CASC4 | Protein CASC4 | 9.046698 | QEDQLQDYRKNNTYLVK |
| P18850 | ATF6 | Cyclic AMP-dependent transcription factor ATF-6 alpha | NFEVS | DHLLLPATTHNKTTRPK |
