# Supplementary material for: Shedding of cancer susceptibility candidate 4 by the convertases PC7/furin unravels a novel secretory protein implicated in cancer progression
Source: Cell Death Dis. 2020 Aug 20;11(8):665. doi: 10.1038/s41419-020-02893-0 (PMC7441151; doi:10.1038/s41419-020-02893-0)
Supplement: Supplementary file 4 — Supplementary Table S3 [file 41419_2020_2893_MOESM4_ESM.docx]

| Protein ID | Gene ID | Protein Name | Ratio glyco-peptide intensity PC7/EV | N-linked glycopeptides  identified |
| --- | --- | --- | --- | --- |
| P78536 | ADAM17 | Disintegrin and metalloproteinase domain-containing protein 17 | 4.92 | SEDIKNVSR |
| Q15758 | ASCT2 | Sodium-dependent neutral amino acid transporter type 2 | 5.79 | SYSTTYEERNITGTR |
| Q15904 | ATP6AP1 | Protein XAP-3 | 5.54 | EIKINASIPAIIIIR |
| Q8NBJ4 | C9orf155 | Golgi membrane protein 1 | 2.04 | IYQDEKAVIVNNITTGER |
| G5E934 | CASC4 | Cancer susceptibility candidate gene 4 protein | NFEVS | QEDQIQDYRKNNTYIVK |
| Q9UBG0 | CLEC13E | C-type lectin domain family 13 member E | 0.69 | WNDSPCNQSIPSICK |
| O75976 | CPD | Carboxypeptidase D | 2.90 | IINTTDVYIIPSINPDGFER |
| O75976 | CPD | Carboxypeptidase D | 2.20 | GYNPVTKNVTVK |
| A9R9N7 | DADB-123D8.3-002 | Major histocompatibility complex, class I, A | 3.25 | GYYNQSEAGSHTVQR |
| O43909 | EXTL1L | Exostosin-like 3 | NFEVS | KSDTQNIIYNVSTGR |
| Q06828 | FM | Collagen-binding 59 kDa protein | 0.68 | IYIDHNNITR |
| P10253 | GAA | 70 kDa lysosomal alpha-glucosidase | 2.29 | GVFITNETGQPIIGK |
| Q99988 | GDF15 | Growth/differentiation factor 15 | 1.21 | IRANQSWEDSNTDIVPAPAVR |
| F6S8M0 | GNS | Glucosamine-6-sulfatase | NFEVS | ASIITGKYPHNHHVVNNTIEGNCSSK |
| Q9Y4L1 | GRP170 | 150 kDa oxygen-regulated protein | NFEVS | DKNGTRAEPPINASASDQGEK |
| E9PDY5 | HS6ST2 | Heparan-sulfate 6-O-sulfotransferase 2 | NFEVS | FVPRYNFTRGDIIR |
| P10809 | HSP60 | 60 kDa chaperonin | NFEVS | VTDAINATR |
| Q92626 | KIAA0230 | Melanoma-associated antigen MG50 | 13.04 | QGEHISNSTSAFSTR |
| P11047 | LAMB2 | Laminin B2 chain | 0.03 | IQRVNNTISSQISR |
| Q13433 | LIV1 | Estrogen-regulated protein LIV-1 | NFEVS | KTNESVSEPR |
| Q9H9K5 | LP9056 | Uncharacterized protein LP9056 | 0.86 | AIINISK |
| Q16549 | PCSK7 | Subtilisin/kexin-like protease PC7 | NFEVS | DINVTGVWER |
| Q16549 | PCSK7 | Subtilisin/kexin-like protease PC7 | NFEVS | RSPGRDINVTGVWER |
| Q16549 | PCSK7 | Subtilisin/kexin-like protease PC7 | NFEVS | SPGRDINVTGVWERNVTGR |
| Q16549 | PCSK7 | Subtilisin/kexin-like protease PC7 | NFEVS | SPGRDINVTGVWER |
| P78395 | MAPE | Melanoma antigen preferentially expressed in tumors | 28.07 | IPTIAKFSPYIGQMINIR |
| P08195-4 | MDU1 | 4F2 cell-surface antigen heavy chain | 5.08 | DIENIKDASSFIAEWQNITK |
| P54802 | NAGLU | Alpha-N-acetylglucosaminidase | 0.68 | SVYNCSGEACR |
| P47972 | NPTX2 | Neuronal pentraxin II | 9.26 | KVAEIEDEKSIIHNETSAHR |
| P02787 | PRO1400 | Beta-1 metal-binding globulin | 2.82 | IIRQQQHIFGSNVTDCSGNFCIFR |
| Q99523 | SORT1 | 100 kDa NT receptor | NFEVS | DITDIINNTFIR |
| P02786 | TFRC | Transferrin receptor protein 1 | 35.88 | KQNNGAFNETIFR |
| P02786 | TFRC | Transferrin receptor protein 1 | NFEVS | DFEDIYTPVNGSIVIVR |
|  |  |  |  |  |
| P51884 | LDC | Keratan sulfate proteoglycan lumican | NFEV | KLHINHNNLTESVGPLPK |
| P02786 | TFRC | Transferrin receptor protein 1 | NFEV | KDFEDLYTPVNGSIVIVR |
| P18850 | ATF6 | Activating transcription factor 6 alpha | NFEV | DHLLLPATTHNKTTRPK |
| Q9H8M5 | ACDP2 | Ancient conserved domain-containing protein 2 | NFEV | VYGQNINNETWSR |
| Q96TA2 | FTSH1 | ATP-dependent metalloprotease FtsH1 | NFEV | SVEIDNKNK |
| Q96J84-2 | KIRREL | Kin of irregular chiasm-like protein 1 | NFEV | IDGGPVILLQAGTPHNLTCR |
| O00461 | GPP130  /GIMPC | Golgi-localized phosphoprotein of 130 kDa | NFEV | KPDPAEQQNVTQVAHSPQGYNTAR |
| E7ETH0 | CFI | C3B/C4B inactivator | NFEV | SIPACVPWSPYIFQPNDTCIVSGWGR |
| G5E934 | CASC4 | Cancer susceptibility candidate gene 4 protein | NFEV | KNNTYLVK |
| P02787 | PRO1400 | Beta-1 metal-binding globulin | NFEV | CGLVPVLAENYNKSDNCEDTPEAGYFAIAVVK |
| P02786 | TFRC | Transferrin receptor protein 1 | NFEV | QNNGAFNETLFR |
| P23142-4 | CTA-941F9.7-002 | Fibulin 1 | NFEV | CATPHGDNASIEATFVK |
