# Supplementary figures and images for: Shedding of cancer susceptibility candidate 4 by the convertases PC7/furin unravels a novel secretory protein implicated in cancer progression
Source: Cell Death Dis. 2020 Aug 20;11(8):665. doi: 10.1038/s41419-020-02893-0 (PMC7441151; doi:10.1038/s41419-020-02893-0)

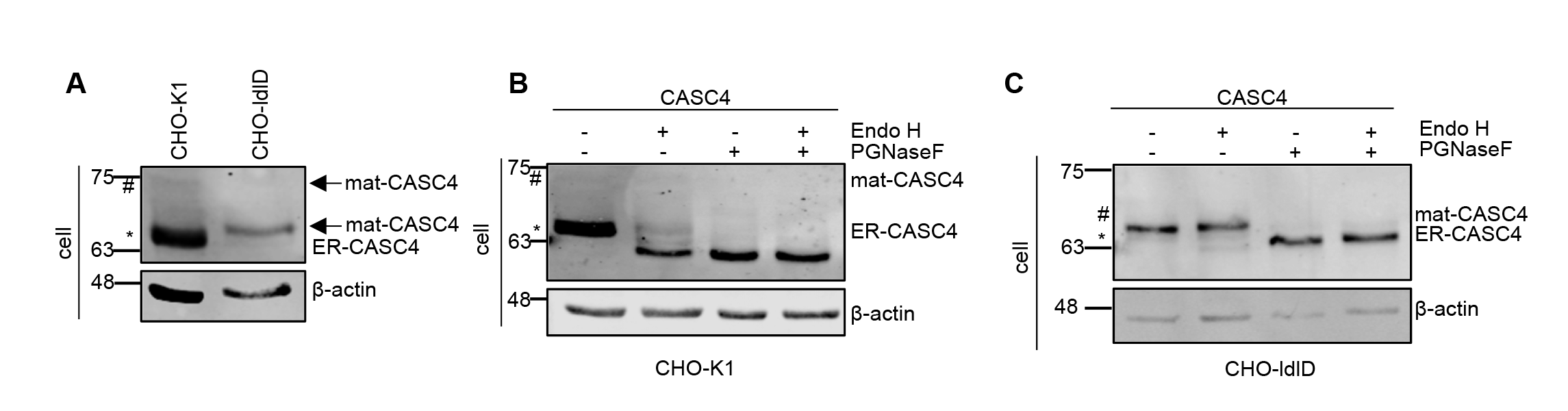

Supplement: Supplementary file 5 — Supplementary Figure S1 [file 41419_2020_2893_MOESM5_ESM.tif]

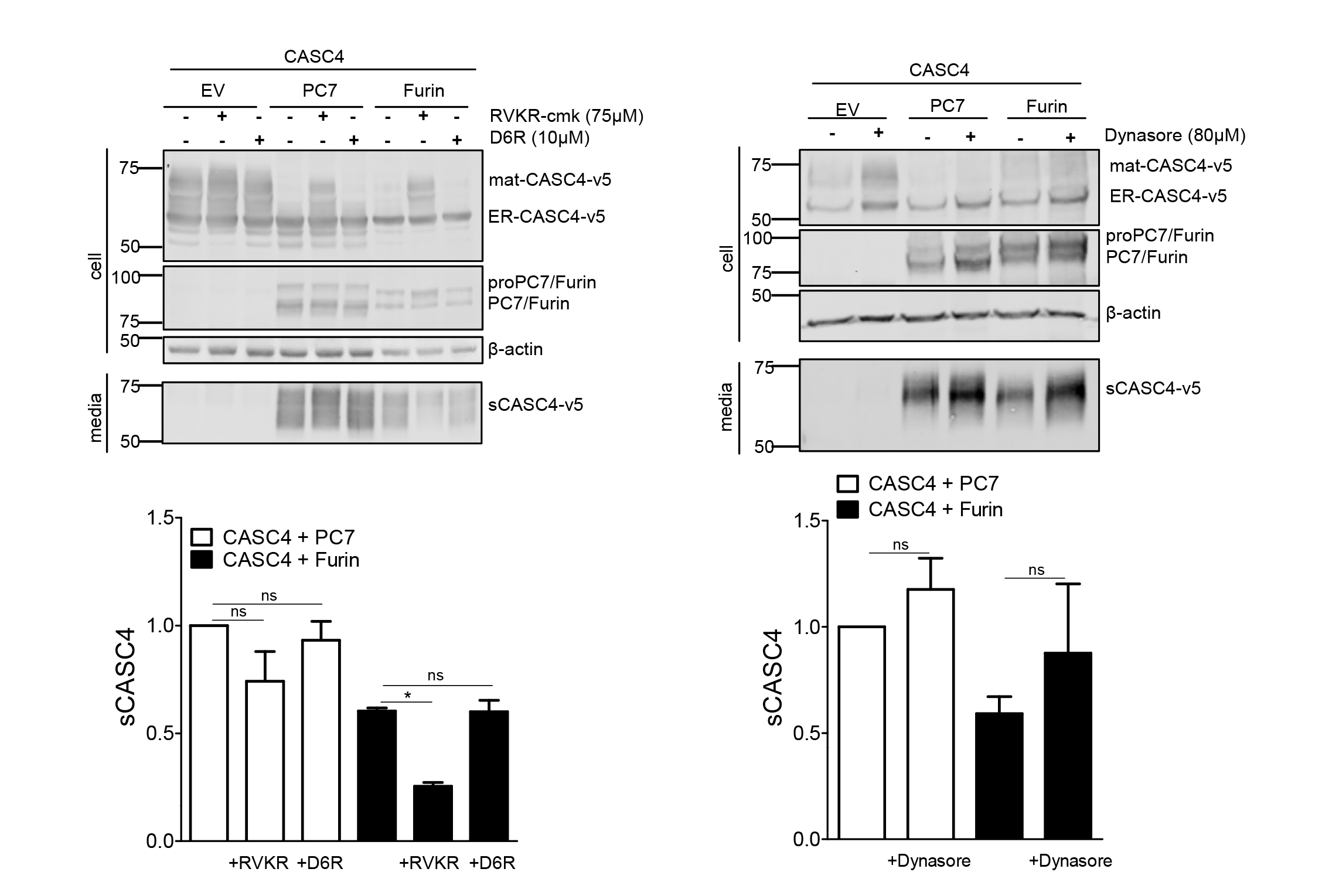

Supplement: Supplementary file 6 — Supplementary Figure S2 [file 41419_2020_2893_MOESM6_ESM.tif]

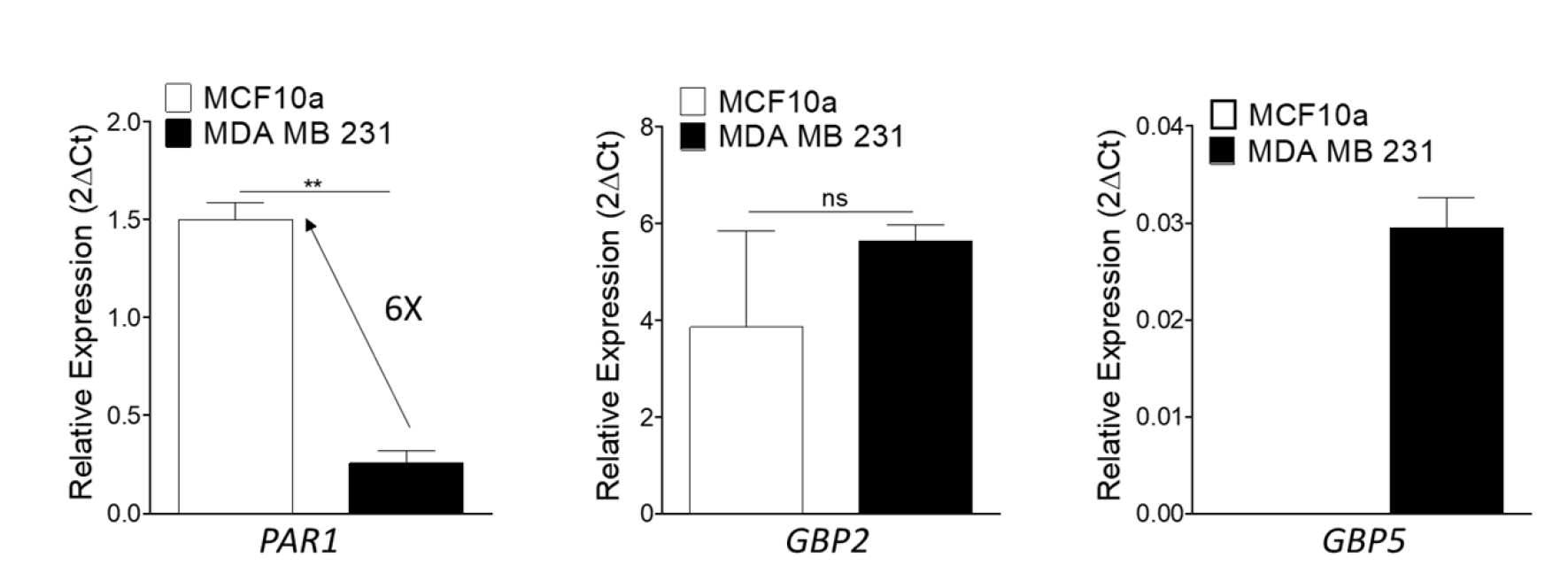

Supplement: Supplementary file 7 — Supplementary Figure S3 [file 41419_2020_2893_MOESM7_ESM.tif]

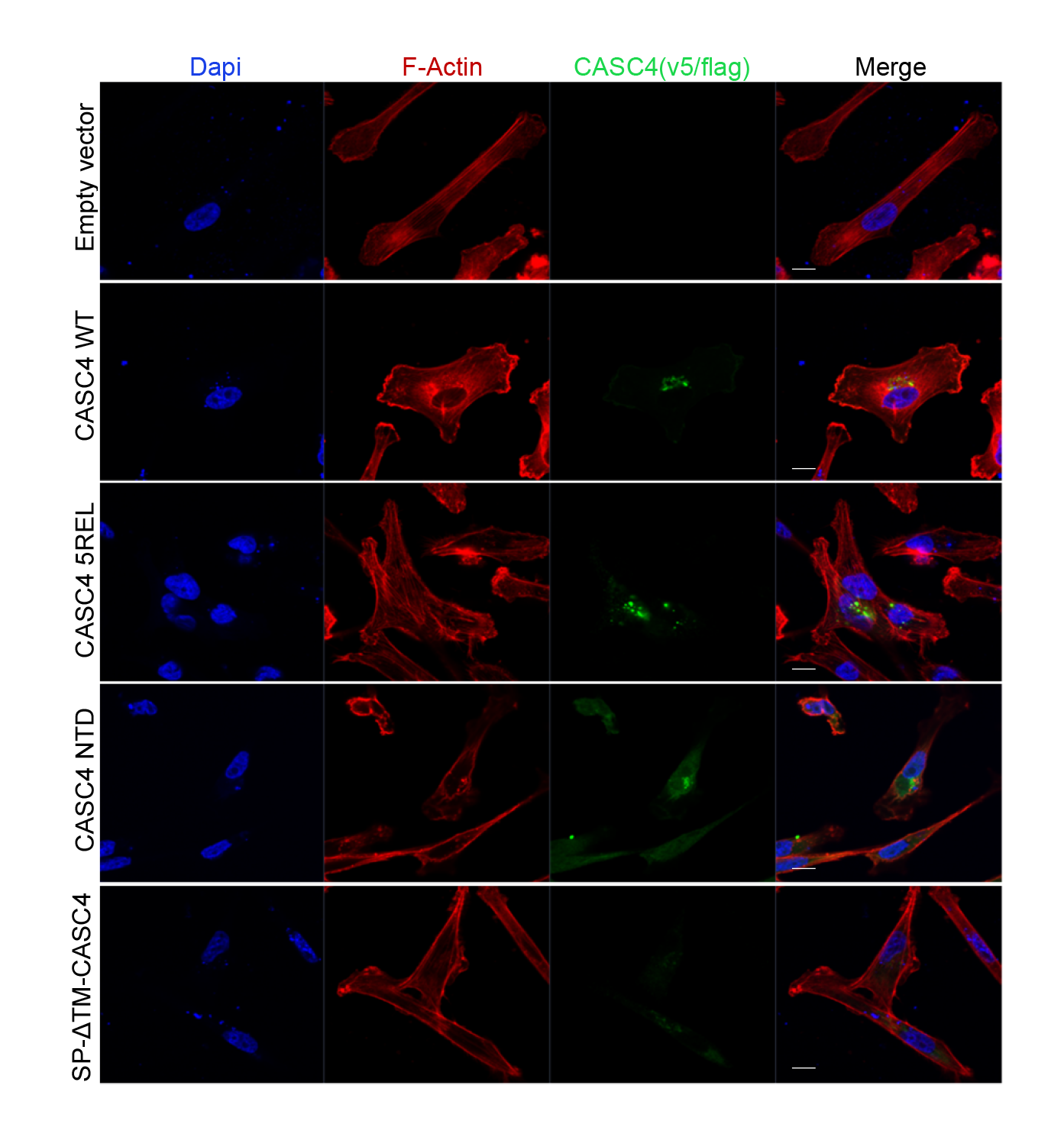

Supplement: Supplementary file 8 — Supplementary Figure S4 [file 41419_2020_2893_MOESM8_ESM.tif]

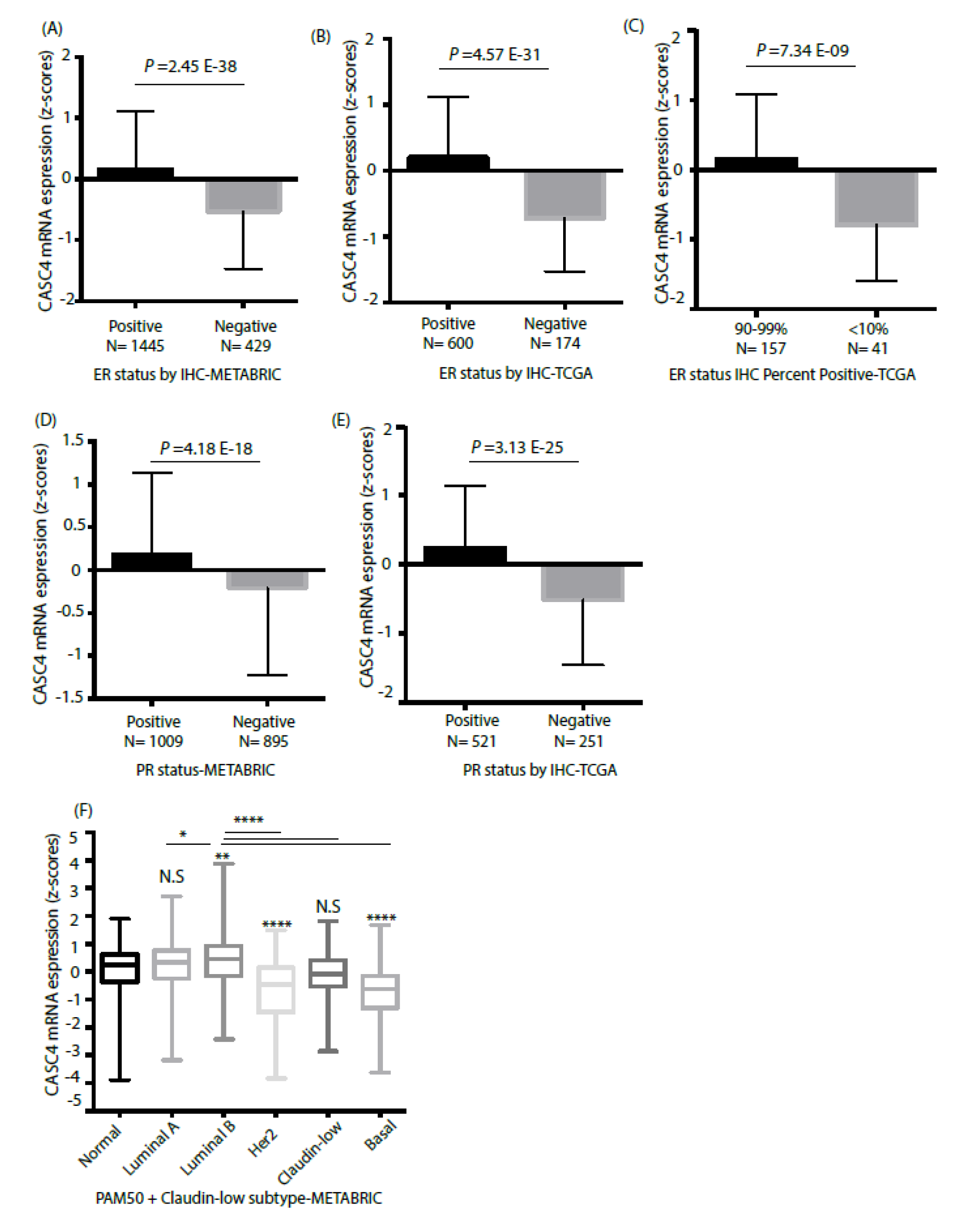

Supplement: Supplementary file 9 — Supplementary Figure S5 [file 41419_2020_2893_MOESM9_ESM.tif]

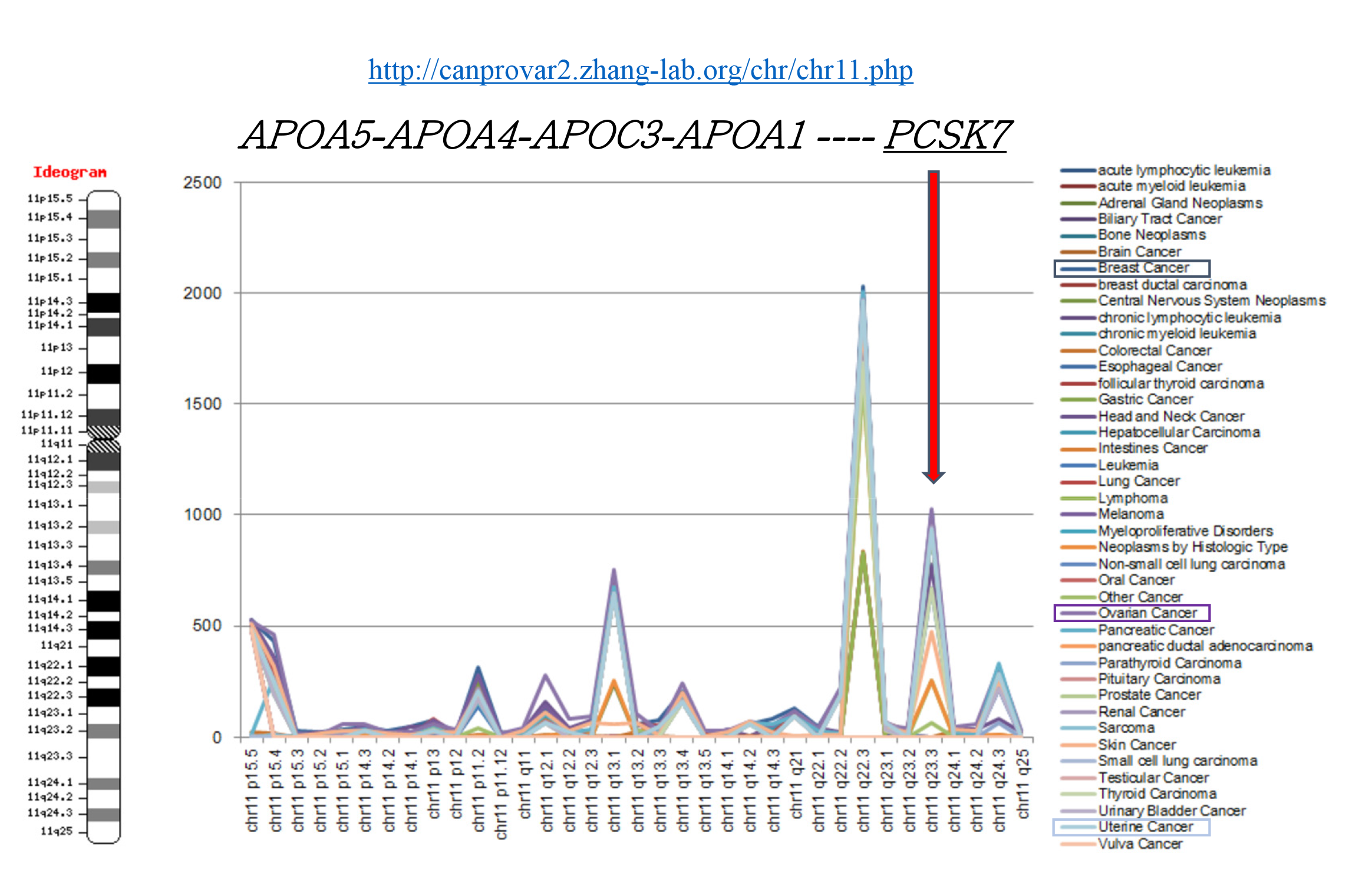

Supplement: Supplementary file 10 — Supplementary Figure S6 [file 41419_2020_2893_MOESM10_ESM.tif]
